# An Integrated Spatial Dynamics—Pharmacokinetic Model Explaining Poor Penetration of Anti-retroviral Drugs in Lymph Nodes

**DOI:** 10.3389/fbioe.2020.00667

**Published:** 2020-06-26

**Authors:** Aditya Jagarapu, Michael J. Piovoso, Ryan Zurakowski

**Affiliations:** ^1^Department of Biomedical Engineering, University of Delaware, Newark, DE, United States; ^2^Department of Electrical and Computer Engineering, University of Delaware, Newark, DE, United States

**Keywords:** HIV, lymph node, PK/PD modeling, sanctuary sites, inflammation, combined anti-retroviral therapy (cART)

## Abstract

Although combined anti-retroviral therapy (cART) suppresses plasma HIV viremia below the limit of detection in a majority of HIV patients, evidence is emerging that the distribution of the anti-retroviral drugs is heterogeneous in tissue. Clinical studies measuring antiretroviral drug concentrations in lymph nodes (LNs) revealed lower concentrations compared to peripheral blood levels suggesting poor drug penetration properties. Our current study is an attempt to understand this poor anti-retroviral drug penetration inside lymph node lobules through integrating known pharmacokinetic and pharmacodynamic (PK/PD) parameters of the anti-retroviral drugs into a spatial model of reaction and transport dynamics within a solid lymph node lobule. Simulated drug penetration values were compared against experimental results whenever available or matched with data that is available for other drugs in a similar class. Our integrated spatial dynamics pharmacokinetic model reproduced the experimentally observed exclusion of antivirals from lymphoid sites. The strongest predictor of drug exclusion from the lymphoid lobule, independent of drug class, was lobule size; large lobules (high inflammation) exhibited high levels of drug exclusion. PK/PD characteristics associated with poor lymphoid penetration include high cellular uptake rates and low intracellular half-lives. To determine whether this exclusion might lead to ongoing replication, target CD4+ T cell, infected CD4+ T cell, free virus, and intracellular IC50 values of anti-retroviral drugs were incorporated into the model. Notably, for median estimates of PK/PD parameters and lobule diameters consistent with low to moderate inflammation, the model predicts no ongoing viral replication, despite substantial exclusion of the drugs from the lymphoid site. Monte-Carlo studies drawn from the prior distributions of the PK/PD parameters predicts increases in site-specific HIV replication in a small fraction of the patient population for lobule diameters greater than 0.2 mm; this fraction increases as the site diameter/ inflammation level increases. The model shows that cART consisting of two nRTIs and one PI is the most likely treatment combination to support formation of a sanctuary site, a finding that is consistent with clinical observations.

## 1. Introduction

Human Immunodeficiency Virus (HIV) is a retrovirus that attacks the CD4 T lymphocytes (CD4 Cells) of the immune system. Combination anti-retroviral (cART) therapy have tremendously reduced HIV associated morbidity and mortality. However, the virus will rebound during treatment interruptions in almost all patients. This is understood to be primarily due to the activation of long-lived, quiescent infected cells that persist during therapy and intermittently activate producing virus.

Recent studies have shown that antiretroviral drugs distribute heterogeneously in various tissues, which raises the possibility that drug concentrations in some tissues may be low enough to allow ongoing HIV replication even in treated patients, forming sanctuary sites. Non-human primate experiments revealed high concentrations of viral RNA and DNA in lymphoid tissues (particularly the spleen, lymph nodes, and gut tissues) compared to suppressed levels in plasma during treatment conditions (North et al., 2009). Pharmacokinetic measurements using positron emission tomography in rats revealed a two-fold decrease in drug concentration in spleen and submandibular lymph nodes, four-fold reduction in mesenteric lymph nodes and the testes, 25-fold reduction in the brain compartment compared to the blood compartment (Di Mascio et al., 2009). Similarly anti-retroviral drug concentrations in human subjects were studied (Fletcher et al., 2014) with multiple sampling of drug concentrations from lymph node, ileum, rectum and plasma compartments after initiation of cART; comparison of the average concentrations in lymph nodes to peripheral blood showed that Tenofovir-diphosphate (TVF-DP), Emtricitabine-triphosphate (FTC-TP), Atazanavir (ATV), Darunavir (DRV), and Efarivenz (EFV) were 80, 66, 100, 99, and 94% lower in the lymph nodes, respectively. These drug distribution studies in animal and human subjects reveal significantly lower drug concentrations in the lymphoid tissues.

Treatment intensification schemes with integrase inhibitor (Raltegravir) revealed a transient increase in 2-LTR circles which are markers of a failed linear DNA integration during viral replication in the host genome (Buzón et al., 2010). Approximately 29% of the HIV positive patients who were on cART with suppressed levels of viral load in peripheral blood compartment observed a transient increase in CD4+ T cells containing HIV 2-LTR following raltegravir intensification. Mathematical modeling on the formation of 2-LTR circles during treatment intensification studies explain that a rapid increase followed by a decrease in 2-LTR circles is evidence of significant levels of ongoing infection, rather than simple virus release from reservoir cells (Luo et al., 2013). This implies the presence of sanctuary sites in these patients.

In our previous work, we proposed a spatial dynamics mathematical model that predicted conditions under which the formation of a sanctuary site is possible inside a lymphoid lobule (Cardozo et al., 2014). Our previous model demonstrated that the 2-LTR dynamics under treatment intensification observed in the INTEGRAL study (Buzón et al., 2010) were possible only if inflammation had increased the size of, and consequently the T cell residence time in, the lymphoid lobule. In the previous study, reduced drug activity within the lobule was assumed, but the mechanisms of drug exclusion were not explored. The current study seeks to estimate the drug penetration for the most commonly used anti-retroviral drugs and understand their transport inside a lymphoid lobule. In order to understand drug transport inside lymph nodes, a model incorporating both reactive and transport mechanisms of cellular components and drugs is developed. Published PK/PD models for representative drugs from each class have been selected and integrated into the spatial dynamic model to evaluate drug penetration inside lymph nodes. Transport between extracellular and intracellular compartments, together with metabolism and degradation rates for each drug, use median published PK/PD parameters. Extracellular drug diffusion rates inside the lymph nodes are calculated using thermodynamic principles, and intracellular transport rates are based on measured T-cell kinetics within lymph nodes as described in our previous work.

## 2. Biological Background

### 2.1. Transport Biology of the Lymph Node

Lymph nodes are surrounded by a fibrous capsule that is contiguous with the afferent and efferent lymphatic ducts, which connect the lymph node to the lymphatic capillary network. Directly under the surface of the fibrous capsule is a network of fluid lymph channels known as the lymphoid sinuses. The sinuses are open fluid channels that form a contiguous fluid path from the afferent lymphatic ducts to the efferent lymphatic ducts, as shown in **Figure 2A**. These are separated from the LN parenchyma by a fenestrated fibrous layer (**Figure 2C**). The parenchyma is subdivided by these fibrous boundaries and sinuses into several functional units called lobules. The lobule interior is densely populated with lymphocytes, which can move freely on a reticular fiber meshwork. The basal end of each lobule extends into the lymph node medulla, where it is adjacent to a large number of narrow sinus channels called the medullary sinuses, which facilitate drainage of the lobule into the sinus network and the efferent lymphatic duct (Willard-Mack, 2006). The entire LN lobule is vascularized by specialized post capillary venous channels called High Endothelial Venules (HEV) illustrated in **Figure 2B**. The HEV, as the name implies, have characteristically thick walls consisting of cuboidal endothelial cells bound by tight junctions. Similar in structure to the capillary walls of the blood-brain barrier, the HEV facilitates highly selective transport between the blood and the lymph node parenchyma.

Lymphocytes in the blood and peripheral tissue enter the LN through one of the two ways: either through specialized post capillary venous channels called High Endothelial Venules (HEV) located in the paracortex region, or through the afferent lymph vessel and the subcapsular sinus. HEV cells express specific adhesion molecules that facilitate efficient transport of the lymphocytes along the endothelial surface of the HEVs. Approximately 2% of the T cells are recruited through HEVs from the recirculating pool per day (Von Andrian and Mempel, 2003). The other cellular components of the lobule, such as macrophages, antigen bearing dendritic cells (DCs) and some lymphocytes, enter from afferent lymphatic vessels, cross the sinus boundaries into the lobule, pass into the medullary sinuses, and eventually leave via the efferent lymphatic vessels.

T cells explore the LN lobule via random walk, and generate an immune response if they encounter antigen presenting cells (APCs) displaying their specific cognate antigen. T cells spend roughly 6–18 h exploring a particular lymph node in uninflamed conditions. However during inflammatory conditions, lymphocyte accumulation is markedly increased and their exit into the efferent lymphatics is transiently blocked (Cahill et al., 1976). This effect increases the probability of lymphocytes encountering presented antigen, by dramatically increasing the time spent exploring the inflamed lymph node. Those lymphocytes that do not encounter cognate antigen will exit the lobule and eventually the LN through the cortical sinus and the efferent lymph vessel (Von Andrian and Mempel, 2003).

#### 2.1.1. Transport of Antiviral Drugs Within a Lymph Node

All antiretroviral drugs used in cART are taken orally, and rapidly transport across the intestinal walls to the bloodstream. The small molecule nature of the drugs facilitate their rapid transport into the lymphoid capillaries. The drugs are taken up into cells via active and passive transport mechanisms, and some of the drugs may undergo metabolic conversion from prodrug to active form. Transport of the drugs and their various metabolites into a lymphoid follicle can thereby occur through two major channels: the blood, through the HEV network, and the fluid lymph, through the sinus network. In both of these channels, the drug may enter the lymph node either as free drug or carried intracellularly by cells migrating into the lymphoid lobule.

#### 2.1.2. cART Mechanisms of Action

HIV infects CD4+ T cells. Uninfected CD4+ T cells are infected by HIV at a mass-action rate forming infected CD4+ T cells. Intracellular HIV events result in the budding of new HIV particles. These infect more CD4 + T cells, continuing the cycle.

Combined anti-retroviral therapy (cART) consists of a combination of drugs that each block one or multiple stages of the viral life-cycle, preventing viral replication. Currently there are six different mechanistic classes of drugs.

HIV initially binds to the CD4 surface receptor and either the CCR5 or CXCR4 co-receptor on the CD4+ T cell. Drugs known as chemokine co-receptor antagonists (CCR5 antagonists) block the virus from binding to the co-receptor and prevent the entry of virus into the host cell (Danjuma, 2009).

After transfer of the viral RNA into the host cytoplasm, the reverse transcriptase enzyme converts the viral RNA into DNA in a process called reverse transcription. Nucleoside Reverse Transcriptase Inhibitor (nRTI) are incorporated into viral DNA instead of natural nucleotides during this stage, resulting in termination of the reverse transcription (Pau and George, 2014). Non-nucleoside Reverse Transcriptase Inhibitors (NNRTI) target and bind to the active catalytic site of the reverse transcriptase (RT) enzyme, preventing the reverse transcriptase enzyme from converting the viral RNA into DNA at the reverse transcription stage (Danjuma, 2009).

Successful reverse transcription produces viral DNA which is transported into the host nucleus for integration into the host genome by the viral integrase enzyme. Integrase strand transfer inhibitors (INSTI) bind to a specific complex between the viral DNA and integrase enzyme, blocking the integration of the viral DNA into the host genome. This results in the formation of episomal artifacts such as linear unintegrated DNA, 1-LTR, and 2-LTR circles which have been investigated as markers for ongoing viral replication (Arts and Hazuda, 2012).

After successful integration into the host genome, transcription and translation results in the production of non-functional polyproteins. The protease enzyme breaks these long chain proteins into functional matrix, capsid and nucleocapsid proteins. Protease Inhibitors (PI) bind to the protease enzyme and prevent the proteolytic cleavage of polyproteins, resulting in the formation of non-infectious viral particles (Arts and Hazuda, 2012).

Three different treatment combinations are most commonly prescribed to treatment-naive patients. Each combination includes two nRTI's which are referred as “backbone” drugs, plus one drug from the PI, INSTI, or NNRTI classes (Eron et al., 2008; Pau and George, 2014).

### 2.2. Integrated Pharmacokinetic-Spatial Dynamics (PKSD) Compartmental Model

#### 2.2.1. Previous Models

Several different groups have introduced lymph node models, all focusing on lymphocyte circulation, migration between blood and lymph, T cell motility inside lymph nodes, and HIV induced immune response during inflammation (Kirschner et al., 2000; Baldazzi et al., 2009; Mirsky et al., 2011; Marinho et al., 2012). None of these focused on drug transport and exclusion or ongoing HIV replication. A recent study modeling persistent viral replication in HIV patients (Lorenzo-Redondo et al., 2016) used a simple two-compartment model assuming heterogeneity in the drug distribution between the two compartments to investigate the possibility of ongoing replication in a drug-privileged node; the simplicity of this model does not allow it to explore mechanisms of drug exclusion from the lymph node.

Our previous work in modeling lymph node consists of a spatial, N - compartmental model (*N*>2) of lymphoid lobules as sanctuary sites explaining viral dynamics in the presence of anti-retroviral drugs. We explored the behavior of these sanctuary sites across a wide range of parameter values and showed that the necessary conditions for low-level ongoing replication is a sanctuary site with large size and low drug efficacy inside it (Cardozo et al., 2014). This study assumed low drug concentrations in the sanctuary sites and did not investigate the mechanism of drug exclusion.

#### 2.2.2. Model Description

In the current study we modified the previous spatial compartmental model to incorporate pharmacokinetic properties of frequently used anti-retroviral drugs. In this work, we model HIV, cell, and drug dynamics in blood, lymphoid sinuses, and lymphoid lobules, including the transport of cells, anti-retroviral drugs and virus between them. Published pharmacokinetic parameters and experimental drug transport values have been used in the model to reflect realistic behavior behind drug transport and their efficacy inside the lobules. Monte-Carlo studies sampling from the published or inferred uncertainty in these parameters has been used to explore the variance and robustness in the behavior. Our results indicate that despite limited drug transport into the lymphoid lobule and resulting low drug efficacy conditions inside the lymphoid lobule, only a small subset of patients on cART will develop the necessary conditions for sanctuary site formation with ongoing HIV replication. The formation of sanctuary sites was far more likely when patients were on nRTI/PI cART compared to nRTI/INSTI or nRTI/NNRTIs cART, and the proportion of patients with ongoing replication increases as the size of the lobules increase.

The model developed in this paper is a reaction/diffusion model. The reaction dynamics describing HIV infection are adapted directly from the basic HIV model (Ho et al., 1995; Wei et al., 1995; Perelson et al., 1997; Nowak and May, 2000; Perelson and Ribeiro, 2013). Published pharmacokinetic studies have been used for modeling the dynamics of anti-retroviral drugs in both plasma and lymphoid lobule (Dixit and Perelson, 2004; Hurwitz et al., 2007; Arab-Alameddine et al., 2012; Habtewold et al., 2017). Modeling assumptions concerning transport of T cells and antiretroviral drugs are as follows:

- Transport of T cells and anti-retroviral drugs between lobule and blood/fluid lymph is assumed to be diffusion-like.
- Transport of T cells and anti-retroviral drugs inside the lobule is assumed to be diffusion-like.
- Free HIV particles are assumed to be blocked from entry into or exit from the lobule. Infected cells may carry HIV in or out.
- Transport between blood and lymphatic sinuses and recirculation within these compartments is assumed to be much faster than transport into and out of the lobule, so blood and lymphatic sinuses are modeled as a single well-stirred compartment.
- Transport between the blood/lymphatic sinus compartment and the lobule occurs primarily at the outer boundary of the lobule. Transport of drugs, lymphocytes and virus across HEV in the lobule interior has been neglected, as most vasculature is associated with the sinus boundaries, and transport of free drugs is expected to be extremely limited across the HEV due to their similarity to the blood-brain barrier (Engelhardt and Wolburg, 2004; Pfeiffer et al., 2008).
- The rate of elimination of the drugs within the lobule compartments is similar to the rate in the blood/lymph compartment.

Based on the above assumptions, the reaction diffusion system has been modeled into a set of compartmental diffusively-coupled ODEs as described in our previous study (Cardozo et al., 2014). The overall system consists of a main compartment that includes blood and the lymphatic sinuses communicating with N spherical domains representing all the lobules in the human body. These lobules are all connected to the blood/lymph compartment, but not to each other. Within each lobule the method of lines have been used to spatially discretize the reaction-diffusion PDE domain into *n*-1 concentric spherical shells, where only the outermost shell is in contact with the blood/lymph compartment. The total number of lobules *N* = 20,000, and the distribution volume of the blood/lymph compartment is 15 liters. Previous work in Cardozo et al. (2014) showed negligible variation in results for compartment numbers larger than 10, so *n* = 10 in this study. Each spherical shell has been denoted by a subscript *s*.

**Figure 1:**
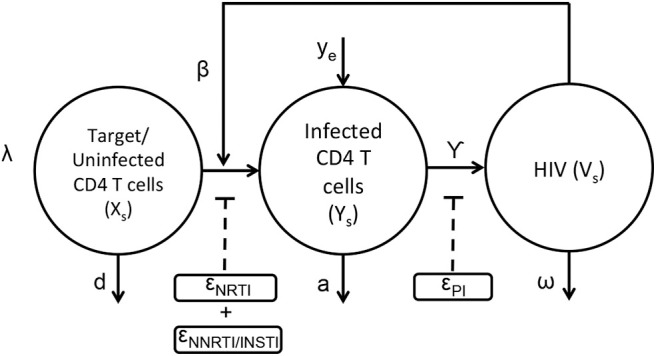
Schematic representation of HIV dynamics in the presence of ART.

The basic viral dynamic model in the presence of anti-retroviral drugs has been previously described (Ho et al., 1995; Wei et al., 1995; Perelson et al., 1997; Nowak and May, 2000; Perelson and Ribeiro, 2013), and is summarized in Figure 1 and Equations (1–3). The states within each compartment *s* are Uninfected/Target CD4+ T cells (**x**_**s**_), actively infected cells (**y**_**s**_), and free virus **v**_**s**_Y_s_). Other viral dynamic parameters include λ, the regeneration rate of healthy T cells, *d*, the turnover rate of healthy T cells, β, the mass-action infection rate of T cells by HIV, *a*, the death rate of productively infected T cells, γ, the production of HIV virus from infected cells, ω, the decay rate of free virus, and *y*_*e*_, the rate at which productively infected cells arise from the quiescent reservoir. These parameter values and their uncertainties have been previously estimated from treatment interruption trial data in Luo et al. (2012), and are detailed in Table S1.

The application of the nRTIs, NNRTIs, INSTIs, and PIs is represented as binary input variables *u*_*n*_,*u*_*nn*_,*u*_*i*_, and *u*_*p*_, respectively. As an example, u_n_=1, u_nn_=1, u_i_=1, u_p_=0 indicates a drug combination consisting of an nRTI,NNRTI and an INSTI excluding PI. Since nRTIs, NNRTIs and INSTIs block the viral replication before viral integration, they reduce infectivity β with efficacies ϵ_*n*_, ϵ_*nn*_, and ϵ_*i*_, respectively. Protease inhibitors reduce the effective virus production rate γ with efficacy ϵ_*p*_. Usually a combination of drugs are used as treatment strategy for HIV (cART) comprising a total of three drugs selecting two from nRTIs and third one from either of the three class i.e., NNRTIs or INSTIs or PIs. The pharmacodynamic values of the drug efficacies are functions of the drug concentrations within the compartment, explained in greater detail in the next section.

(1) $$\begin{array}{l} \dot{\text{x}}=\lambda -d\text{x}-\beta \text{x}\text{v}\left(1-u_{n}\epsilon_{n}\right)\left(1-u_{nn}\epsilon_{nn}\right)\left(1-u_{i}\epsilon_{i}\right) \end{array}$$

(2) $$\begin{array}{l} \dot{\text{y}}=\beta \text{x}\text{v}\left(1-u_{n}\epsilon_{n}\right)\left(1-u_{nn}\epsilon_{nn}\right)\left(1-u_{i}\epsilon_{i}\right)-a\text{y}+y_{e} \end{array}$$

(3) $$\begin{array}{l} \dot{v}=\gamma \left(1-u_{p}\epsilon_{p}\right)\text{y}-\omega \text{v} \end{array}$$

**Figure 2:**
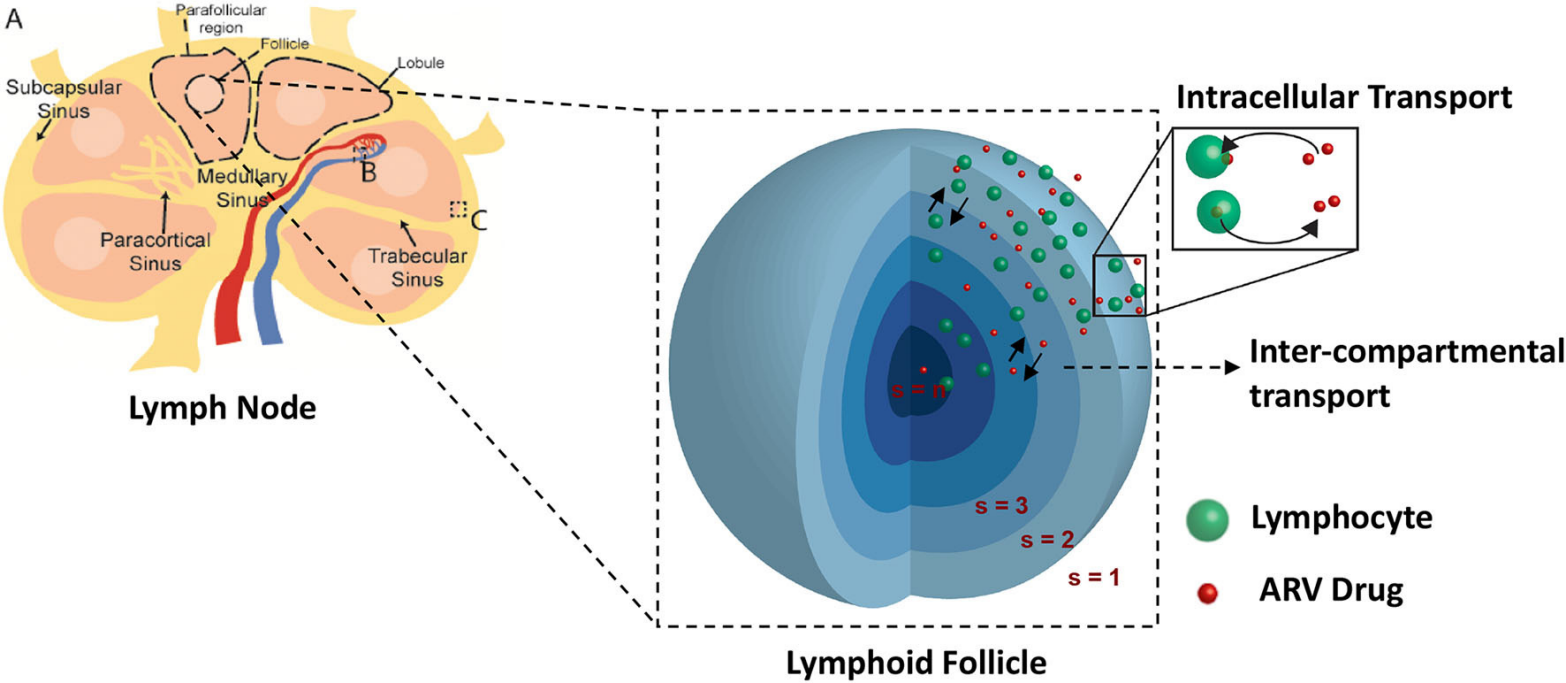
Spatial compartmental model. Lymph node diagram **(A)** highlights the vascular interface **(B)**, and sinus interface **(C)**.

The HIV infection dynamics are reaction dynamics occurring between species in the same spatial compartment. Species also migrate between compartments following diffusion principles. The transport of lymphocytes, ARV drugs and HIV between compartments is shown in Figure 2. Compartment (*s* = 1) consisting of the blood and fluid lymph, is in contact with only the outermost shell (*s* = 2) of the lymphoid lobules which is further linked with other *n*−1 compartments in series as shown in Figure 2. Transport of cellular and molecular components between these compartments depends on their diffusive properties and their concentration differences between any two compartments. Equations (4–9) are the ODE equations resulting from the method of lines discretization of the reaction diffusion equations, including spatial transport mechanisms along with the HIV dynamics. Equations (4–6) represent transport between the blood/lymph (*s* = 1) with the outermost compartment (*s* = 2) of the lobule as discussed above. The rate of diffusive flux is directly proportional to concentration difference, surface area and inversely proportional to the volume and length between any two adjacent compartments. The set of indices for the compartments which are adjacent to compartment s is the set ψ_*s*_. *A*_(*i, s*)_, represents the surface area between any two adjacent compartments *i* and *s* in the lobule. *V*_*s*_,$\frac{D_{\left(xi,s\right)}}{l}=\frac{D_{\left(yi,s\right)}}{l}$, $\frac{D_{\left(vi,s\right)}}{l}$ represents the volume, effective diffusivity of uninfected, infected CD4 T cells and virions of the layer between the *i*^*th*^ and *s*^*th*^ compartments. In this case, cART consisting of two NRTIs and a PI is modeled. The efficacies of the two NRTIs and the PI have been denoted as “ϵ_NRTI1_,” “ϵ_NRTI2_,” “ϵ_PI_.” The actual effectiveness is a function of the concentration of the drug within the compartment and the pharmacodynamics of the drug, which are discussed in the next section.

(4) $$\begin{array}{l} {\dot{\text{x}}}_{1}=\lambda -d{\text{x}}_{1}-\beta {\text{x}}_{1}{\text{v}}_{1}\left(1-\epsilon_{NRTI1,1}\right)\left(1-\epsilon_{NRTI2,1}\right)\\ +\;N\frac{D_{x1,2}}{l}\frac{A_{1,2}}{V_{1}}\left({\text{x}}_{2}-{\text{x}}_{1}\right) \end{array}$$

(5) $$\begin{array}{l} {\dot{\text{y}}}_{1}=\beta {\text{x}}_{1}{\text{v}}_{1}\left(1-\epsilon_{NRTI1,1}\right)\left(1-\epsilon_{NRTI2,1}\right)-a{\text{y}}_{1}+y_{e}\\ +\;N\frac{D_{y1,2}}{l}\frac{A_{1,2}}{V_{1}}\left({\text{y}}_{2}-{\text{y}}_{1}\right) \end{array}$$

(6) $$\begin{array}{l} {\dot{\text{v}}}_{1}=\gamma \left(1-\epsilon_{PI,1}\right){\text{y}}_{1}-\omega {\text{v}}_{1}\\ +\;N\frac{D_{v1,2}}{l}\frac{A_{1,2}}{V_{1}}\left({\text{v}}_{2}-{\text{v}}_{1}\right) \end{array}$$

Transport between adjacent compartments within the lobule is described in Equations (7–9).

(7) $$\begin{array}{l} {\dot{\text{x}}}_{s}=\lambda -d{\text{x}}_{s}-\beta {\text{x}}_{s}{\text{v}}_{s}\left(1-\epsilon_{NRTI1,s}\right)\left(1-\epsilon_{NRTI2,s}\right)\\ +\underset{i\in \psi_{s}}{\sum }\frac{D_{xi,s}}{l}\frac{A_{i,s}}{V_{s}}\left({\text{x}}_{i}-{\text{x}}_{s}\right) \end{array}$$

(8) $$\begin{array}{l} {\dot{\text{y}}}_{s}=\beta {\text{x}}_{s}{\text{v}}_{s}\left(1-\epsilon_{NRTI1,s}\right)\left(1-\epsilon_{NRTI2,s}\right)-a{\text{y}}_{s}+y_{e}\\ +\underset{i\in \psi_{s}}{\sum }\frac{D_{yi,s}}{l}\frac{A_{i,s}}{V_{s}}\left({\text{y}}_{i}-{\text{y}}_{s}\right) \end{array}$$

(9) $$\begin{array}{l} {\dot{\text{v}}}_{s}=\gamma \left(1-\epsilon_{PI,s}\right){\text{y}}_{s}-\omega {\text{v}}_{s}+\underset{i\in \psi_{s}}{\sum }\frac{D_{vi,s}}{l}\frac{A_{i,s}}{V_{s}}\left({\text{v}}_{i}-{\text{v}}_{s}\right) \end{array}$$

#### 2.2.3. Pharmacokinetic/Pharmacodynamic (PK/PD) Models

Efficacy (pharmacodynamics) for each drug is assumed to follow Hill dynamics as described in Equation (10). The terms IDC(t), IC_50_ and “*n*_*drug*_” in Equation (10) denote the effective drug concentration (usually the intracellular concentration of the active form) at any time “t,” amount of drug concentration required to produce a 50% inhibitory effect and the Hill coefficient for the drug, respectively. Drugs from each class were chosen based on the availability of published PK/PD models and their associated parameters. The following sub-sections describe the PK/PD models for the drugs used in the current study.

(10) $$\in_{\text{drug}}=IDC{\left(t\right)}^{n_{drug}}/\left(IC_{50}^{n_{drug}}+IDC{\left(t\right)}^{n_{drug}}\right)$$

##### 2.2.3.1. NRTI: nucleoside reverse transcriptase inhibitor (Tenofovir, Lamivudine)

The most commonly used nRTIs are tenofovir and abacavir, both of which are used in combinations with emtricitabine or lamivudine as the second nRTI. In our current simulations, we chose to use tenofovir and lamivudine as the two nRTI drugs in the antiretroviral repertoire. The intracellular pharmacokinetic models for these drugs have been adopted from Baheti et al. (2011), Dixit and Perelson (2004), and Hurwitz et al. (2007).

**Figure 3:**
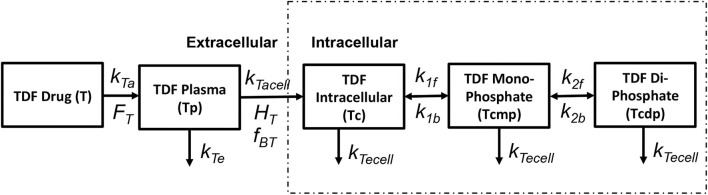
Tenofovir pharmacokinetic model (Dixit and Perelson, 2004).

Tenofovir is usually administered in its monophosphorylated analog Tenofovir Disoproxil Fumarate (TDF) in doses of 300 mg/day. The pharmacokinetics of tenofovir are shown in Figure 3. After oral administration it is rapidly adsorbed into the plasma at a rate k_Ta_ with a bioavailability F_T_ and eliminated from the plasma compartment(T_p_) at a rate k_Te_. TDF binds minimally with the proteins by a factor f_BT_ in the extracellular space and starts to accumulate in the intracellular compartment at a rate k_Tacell_ across the cell boundary with a partition coefficient H_T_. Once the monophosphate form of the drug reaches the intracellular space, it undergoes only two steps of phosphorylation to obtain the triphosphate anabolite unlike other nRTIs that undergo three steps of phosphorylation. The forward rate constants for the formation of TDF monophosphate and TDF diphosphate are k_1f_ and k_2f_ while the backward rate constants are k_1b_ and k_2b_, respectively. All the intracellular components of the drug are eliminated at a rate k_Tecell_ from the cell. The intracellular concentrations of TDF, TDF monophosphate and TDF diphosphate are represented as T_c_,T_cmp_, and T_cdp_, respectively.

**Figure 4:**
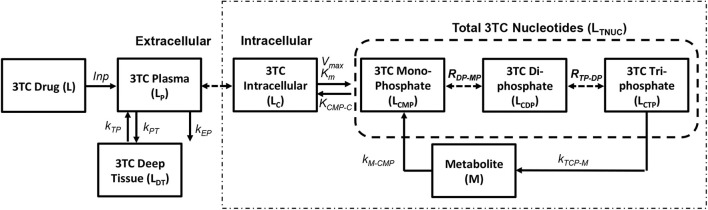
Lamivudine pharmacokinetic model (Hurwitz et al., 2007).

The pharmacokinetic model for simulating lamivudine (3TC) has been adopted from Hurwitz et al. (2007) as shown in Figure 4. The extracellular pharmacokinetics have been described by a two compartment model (Plasma and Deep tissue), where the drug absorption into plasma was assumed to be a zero order process with input (F*D/T_1_) where “F” is the bioavailability of the drug, “D” is the drug dosage (150 mg twice daily) and “T_1_” is the time period (1 or 3 h) for the zero order absorption. The plasma concentration (L_P_) is further distributed between the deep tissue (L_DT_) and the intracellular compartments (L_C_). Drug elimination from the plasma compartment takes place at a rate k_EP_. The inter-compartmental clearance rates k_PT_ and k_TP_ describe the rate at which the plasma concentration is transferred from plasma to tissue and vice versa. Rapid equilibrium is assumed to be achieved between the plasma concentration and intracellular concentration of 3TC due to action of equilibrative nucleoside transporters present on the cell membranes of lymphocytes as assumed in Hurwitz et al. (2007). Intracellular 3TC undergoes series of phosphorylation steps to form 3TC triphosphate (L_CTP_). Formation of 3TC monophosphate (L_CMP_) was assumed to be rate limiting and the conversion was modeled using Michaelis-Menten reaction with maximum rate (V_m_) and Michaelis-Menten constant (K_M_). Rapid equilibrium is assumed in-between phosphorylation steps with ratios R_DP/MP_ and R_TP/DP_ relating the concentrations between the 3TC-diphosphate to 3TC-monophosphate and 3TC-triphosphate to 3TC-diphosphate. Re-circulation of 3TC-triphosphate to 3TC-monophosphate was also considered with the formation of an intermediate metabolite (M) with K_CTP−M_ as the rate of formation and K_M−CMP_ as the rate of conversion of metabolite to 3TC-monophosphate.

##### 2.2.3.2. NNRTI: non-nucleoside reverse transcriptase inhibitor (Efarvirenz)

**Figure 5:**
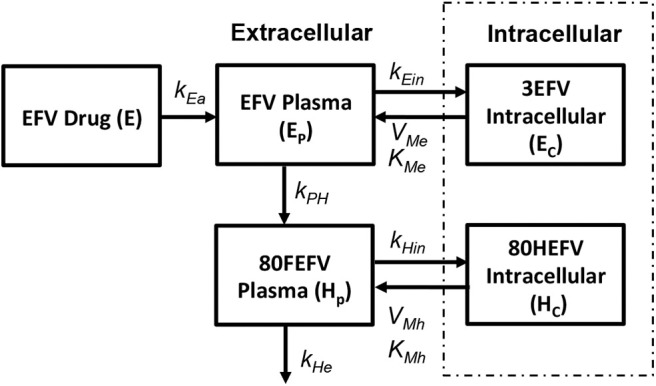
Efarivenz pharmacokinetic model (Habtewold et al., 2017).

Currently five different NNRTIs have been approved by FDA for antiretroviral therapy. For our current study we chose to use efarivenz due to the availability of a previously published intracellular pharmacokinetic model by Habtewold et al. (2017) as shown in Figure 5. Efarivenz (EFV) is usually prescribed once daily in doses of 600mg single pill. The pharmacokinetic model was described by a two-compartmental model with concentrations of EFV distributed between the plasma and peripheral blood mono-nuclear cells (PBMC). First order kinetics has been used to describe the transfer of drug from gastrointestinal tract (E) to plasma (E_p_) at a rate k_Ea_ and from plasma (E_p_) to the intracellular compartment (E_c_) at a rate k_Ein_. Transport of drug from intracellular compartment (E_c_) to plasma (E_p_) has been assumed to follow a nonlinear saturating inter-compartmental clearance process with V_Me_ and K_Me_ as the maximum rate and Michaelis-Menten constant, respectively. EFV in plasma is further converted into its metabolite 80HEFV (H_p_) at a rate k_Ee_ and the inter-compartmental clearance of 80HEFV was modeled with similar kinetics for forward and backward transport of EFV between plasma (H_p_) and intracellular compartments (H_c_). NNRTIs do not require phosphorylation like NRTIs to inhibit reverse transcriptase. Hence we used the concentration of EFV in the intracellular compartment to evaluate the instantaneous drug efficacy in Equation (10) for the viral dynamics in our reaction-diffusion model.

##### 2.2.3.3. INSTI: integrase inhibitor (Raltegravir)

**Figure 6:**
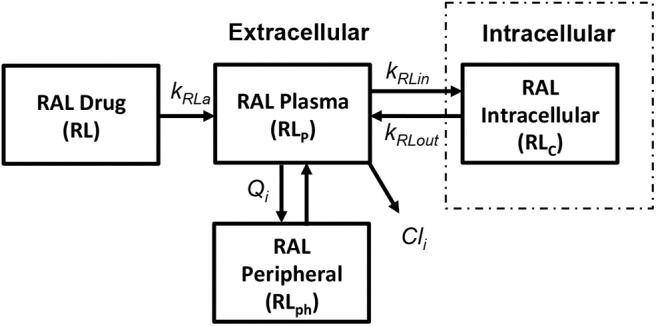
Raltegravir pharmacokinetic model (Arab-Alameddine et al., 2012).

We use the integrase inhibitor Raltegravir (RAL) in this study; its pharmacokinetics were studied in both HIV-positive (HIV^+^) and healthy individuals in Arab-Alameddine et al. (2012). A basic two compartmental model with first order absorption rate (k_Ra_) from the gastrointestinal tract (R) to plasma (R_p_) and with an inter-compartmental clearance (Q_i_) between plasma (R_p_) and peripheral compartment (R_ph_) was described in their study as shown in Figure 6. The other parameters that were estimated in this population pharmacokinetic model include the apparent volumes of distribution for plasma (V_p_) and peripheral compartment (V_ph_) along with apparent clearance of drug from the plasma compartment (Cl_i_). The above pharmacokinetic study did not evaluate the intracellular pharmacokinetics in their model. However, other studies estimated the cellular penetration values (ration of raltegravir concentration between intracellular and plasma compartments) as between 5% (Fayet Mello et al., 2011) to 11% (Wang et al., 2011). Using this range of penetration values, we assumed steady state conditions between plasma (R_p_) and intracellular compartments (R_c_) to estimate the forward (k_Rin_) and backward (k_Rout_) drug transfer constants across the cellular membrane for raltegravir. Recommended dosage of 400 mg twice daily for raltegravir has been used in our simulations.

##### 2.2.3.4. PI: protease inhibitor (Ritonavir)

**Figure 7:**
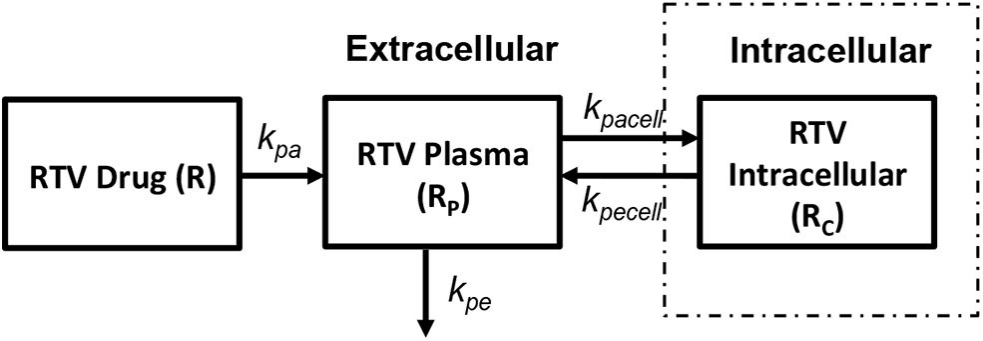
Ritonavir pharmacokinetic model (Dixit and Perelson, 2004).

In order to obtain the intracellular drug concentrations for PI we chose to adopt the pharmacokinetic model on ritonavir used by Dixit and Perelson (2004) to evaluate its effect as monotherapy in viral dynamics, shown in Figure 7. A relatively simple model of drug transport from plasma (R_p_) to intracellular compartment (R_c_) has been discussed with k_pa_, k_pe_, k_pacell_, k_pecell_ describing the rate of drug absorption from the drug compartment (R), rate of drug elimination from plasma compartment, rate of forward transport and backward transport across the cellular membrane, respectively. *In vitro* studies suggest that the intracellular concentrations of ritonavir reach steady state very quickly describing that the cell membrane offers little resistance for ritonavir transport. However, the steady state concentrations are different across the cellular membrane which can be modeled using a non-unit partition coefficient. The non-unit partition coefficient was established from *in vitro* studies in Dixit and Perelson (2004) and included the protein binding fraction to estimate the concentration of intracellular ritonavir concentration as a ratio of the plasma concentration. We modified this transfer coefficient (partition coefficient along with protein binding effect) into a forward (k_pacell_) and backward (k_pecell_) rate constants by adjusting the ratios such that they give the same transfer coefficient as used in Dixit and Perelson (2004). This method was adopted to evaluate the time evolution of the intracellular ritonavir concentration for our simulations. Six hundred milligrams of pill with twice daily as dosage regimen has been chosen to evaluate the time evolution of the plasma and intracellular concentrations as used in Dixit and Perelson (2004).

### 2.3. **Parameter Values and Uncertainties**

All the parameters used in this study have been obtained from previously published works on HIV viral dynamics, experimental studies on drug transport and T cell motion inside the lymph node and population pharmacokinetic studies on drug distribution and metabolism in HIV patients. These parameters are known to have a significant degree of within-patient drift and between-patient variability. To investigate the range of behaviors consistent with the parameter heterogeneity, we undertook a Monte-Carlo analysis drawing from prior distributions for each uncertain parameter. For parameters that were published with experimental uncertainty intervals, we have used the published uncertainty values; for parameters without published uncertainty, we have imputed an uncertainty interval of ±20% of the published nominal value. The HIV dynamic parameters are highly correlated, and drawing independently from each parameter's prior can result in non-physiological behavior. Instead, we have drawn our HIV dynamic parameter values from the multi-dimensional distributions obtained by Bayesian model fits to interruption trial data from 12 HIV patients previously published in Luo et al. (2012). The exact parameter values used in this study, together with the uncertainty intervals used in the Monte-Carlo studies, can be found in Tables S1–S8.

#### 2.3.1. Viral Dynamics

Parameters for viral dynamics have been obtained from parameter identification studies for HIV sampled from frequently sampled viral load data from ten patients enrolled in the published AutoVac HAART interruption study (Ruiz et al., 2000). The viral dynamic parameters have been estimated using a Bayesian Markov-Chain Monte-Carlo method. The posterior estimates on the parameters are based on the experimental data of HIV patients who had 3–5 treatment interruption cycles (Luo et al., 2012). The estimated parameters with confidence intervals that were used in the current study can be found in Table S1.

#### 2.3.2. Diffusion Parameters

##### 2.3.2.1. Effective diffusivity of T cells and virus

The effective diffusivity for T cells across the boundary between lymphoid lobule and the blood/lymph compartment is estimated as described in our previous work (Cardozo et al., 2014). Previous experimental studies have shown that lymph nodes with an average diameter of 1 mm in a mouse recruit approximately 2% of the circulating T cells in the absence of infection. Hence, the effective diffusivity of T cells across the boundary i.e., between the blood compartment and the outermost spherical compartment of the lymphoid lobule D_x_b,LN__/l, D_y_b,LN__/l can be obtained from the equation (D_x_b,LN__/l)(A_b,LN_/V_b,LN_)x_b_ = 0.02x_b_ where A_b,LN_, V_b,LN_ are the area and volume of the lymphoid lobule. The effective diffusivity D_x_b,LN__/l equals 1/300 mm/day when the lymph node diameter is 1 mm. Since the target CD4 cells and infected CD4 cells have similar effective diffusivity, the calculated values for D_x_b,LN__/l = D_y_b,LN__/l.

The effective diffusivity within the lymphoid lobule (i.e., between any two concentric compartments in our model) is equal to the average value of the experimentally observed motility coefficient of T-cells within lymphoid lobules which is 0.1 mm^2^/day (Von Andrian and Mempel, 2003; Beltman et al., 2007; Mirsky et al., 2011; Girard et al., 2012) divided by the length of each layer l = *r*/(*n*-1), where “r” is the radius of the lymphoid lobule and “n” is the total number of compartments in the model.

##### 2.3.2.2. Effective diffusivity of drugs

Effective diffusivity of the drugs inside the lymphoid lobule has been calculated using the diffusion coefficients theoretically obtained from the Einstein-Stokes equation and the viscosity of fluid lymph. Effective viscosity within the lymph node will be higher due to the high density of cells and extracellular matrix components. Experiments tracking the motility of single-molecule chemokine AF647-tagged CXCL13 using high speed light microscopy system capable of millisecond sampling in an *ex vivo* native mouse lymph node environment allow for direct measurement of these values (Miller et al., 2018). The experimentally observed values were 22.7 times less than the values for fluid lymph; we adjusted our values by the same factor. The adjusted drug diffusion coefficients used in our simulations can be found in Table S2.

In order to evaluate the effective drug diffusivity across the boundary i.e., between the blood/ fluid lymph and lymphoid lobule, we calculate the ratio of effective diffusivity values for T cells between the boundary and the inner lobule (values discussed in the previous section) and assume a similar ratio for effective diffusivities across the boundary and inner lobule for the antiretroviral drugs. Hence, we multiply this ratio (boundary/inner lobule for T cells) with the adjusted drug diffusivity inside the lymphoid lobule (obtained from Einstein stokes equation) to obtain the effective diffusivity across the boundary i.e., between blood/lymph and the lobule. For an average lymphoid lobule with a diameter of 0.2 mm with 10 total compartments, i.e., *n* = 10, the effective diffusivity (D/l) would be 9 mm/day. The ratio of effective diffusivity at the boundary to the inner lobule would be 1/2,700 which was used to estimate the effective diffusivity for the drugs at the boundary (Table S3). Recall that we are assuming that transport for most species is dominated by transport from the subcapsular sinus, which is separated from the lobule by a fibrous epithelial boundary—this ratio can be interpreted as the fraction of total surface area available for transport across this boundary.

#### 2.3.3. Pharmacokinetic Parameters

All the reaction rate constants, elimination rate constants for each individual drug have been obtained from the published pharmacokinetic studies as mentioned above. Parameters for evaluating the instantaneous efficacy of the drug such as IC_50_ and hill coefficient “*n*,” have been determined from the dose-response curves on antiretroviral drugs studied by Shen et al. (2008). Pharmacokinetic parameter estimates used in our current study can be found in Tables S4–S8.

### 2.4. **Monte-Carlo Simulations**

Integrating the above discussed population pharmacokinetic models along with the spatial compartmental model gives us the integrated pharmacokinetic spatial compartmental model to understand the drug transport and viral dynamics inside the lymphoid lobule of a HIV patient. In order to investigate the robustness of drug transport effects to parameter uncertainty and inter-patient variability, we employed Monte Carlo simulations by sampling random values from parameter distributions on viral dynamics and pharmacokinetics. The 95% confidence intervals from which we draw our Monte-Carlo samples are shown in the Supplementary Tables. Simulations were carried out on varying sizes of lymphoid lobule with diameters of 0.01, 0.05, 0.10, 0.20, 0.35, and 0.50 mm. Five thousand simulations were carried out on each diameter of the lobule under each of three treatment conditions, with cART consisting of two nRTIs as the backbone drugs along with either a PI, INSTI or an NNRTI. Simulations were carried out for a time period of 100 days, which was long enough to reach steady state.

## 3. Results

### 3.1. Drug Penetration vs. Lobule Diameter

**Figure 8:**
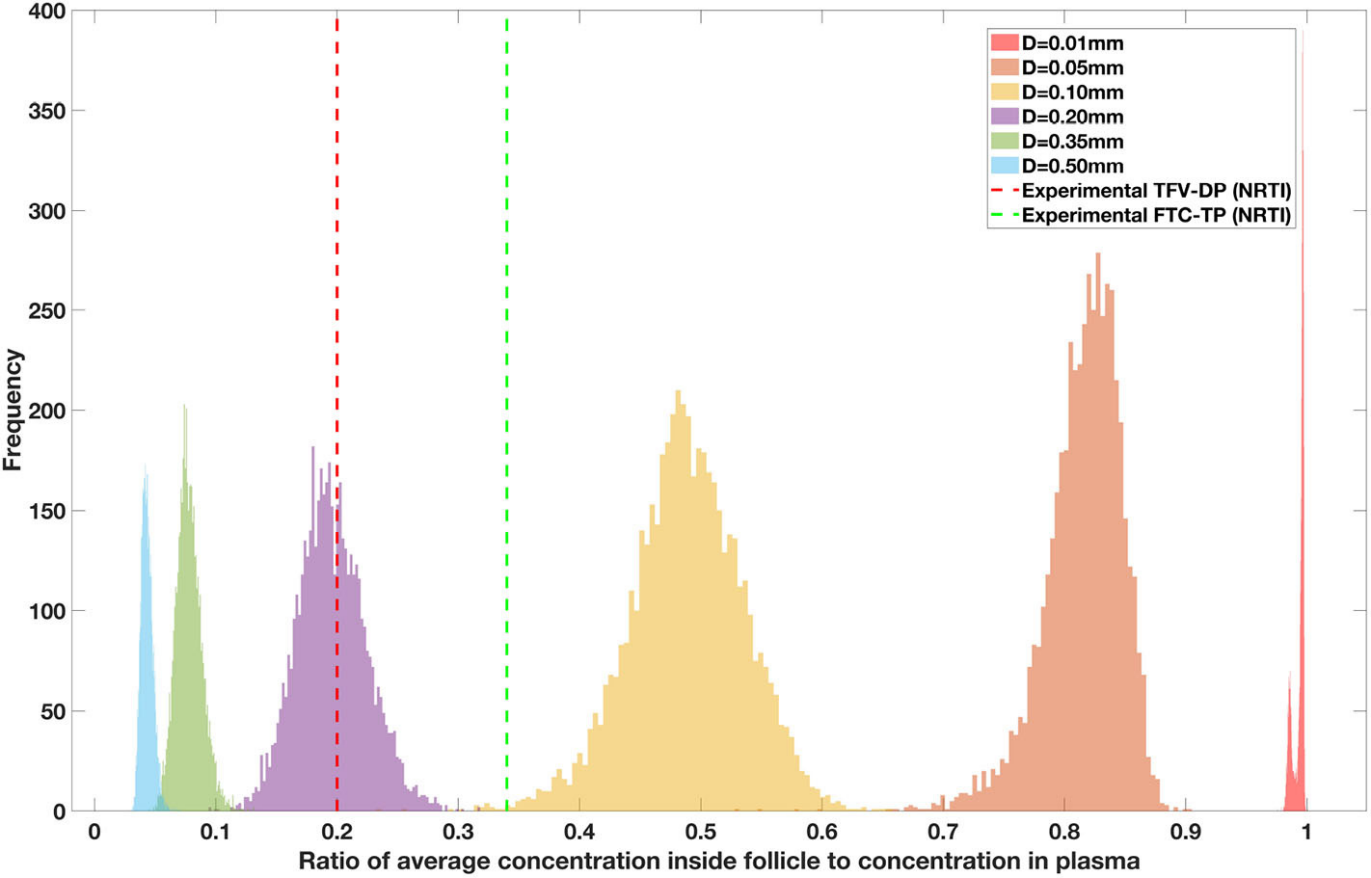
Concentration ratio (lobule/plasma) of Tenofovir-diphosphate, TFV-DP (nRTI) with change in lobule diameter.

**Figure 9:**
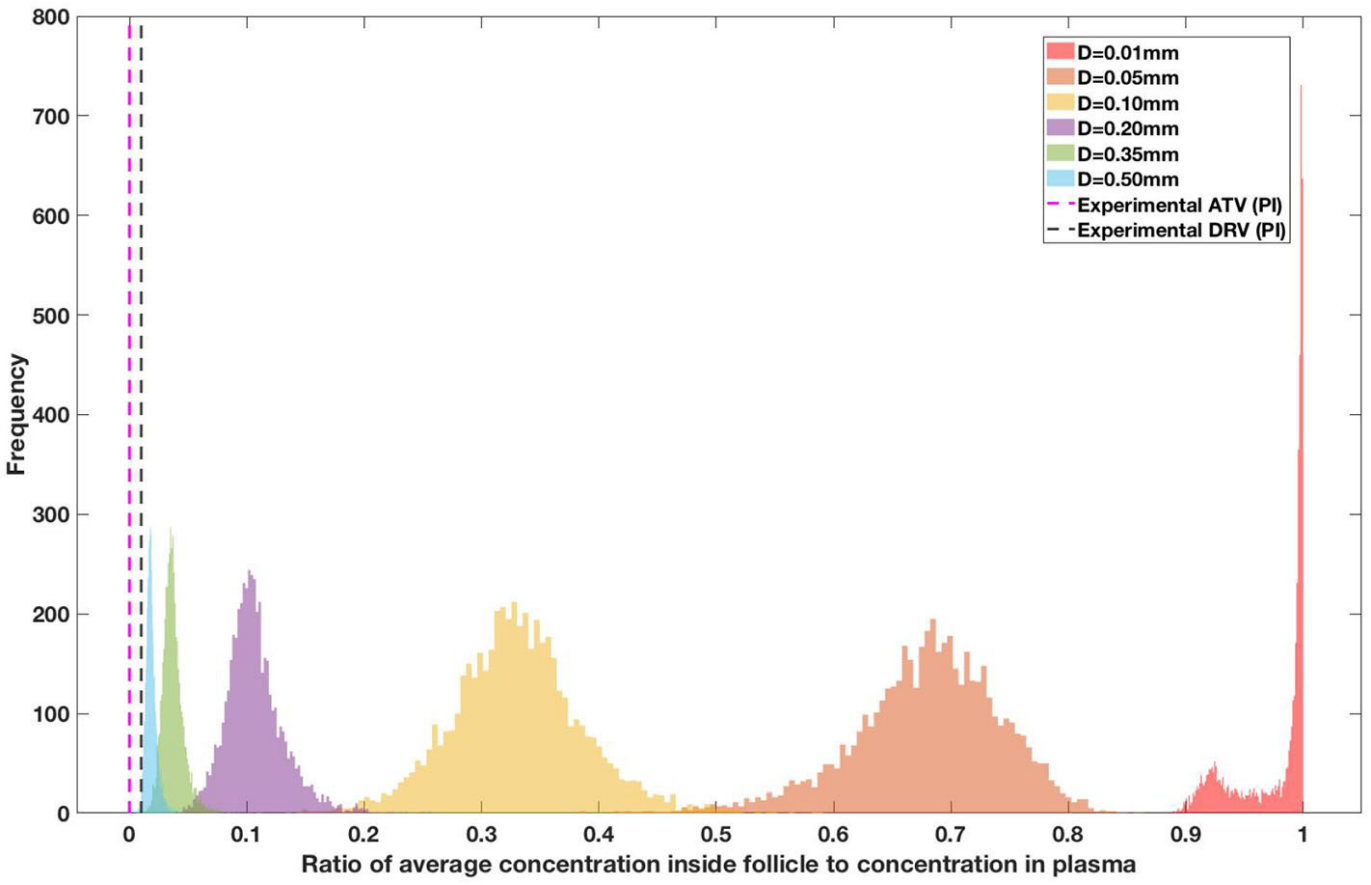
Concentration ratio (lobule/plasma) of Ritonavir, RTV (PI) with change in lobule diameter.

**Figure 10:**
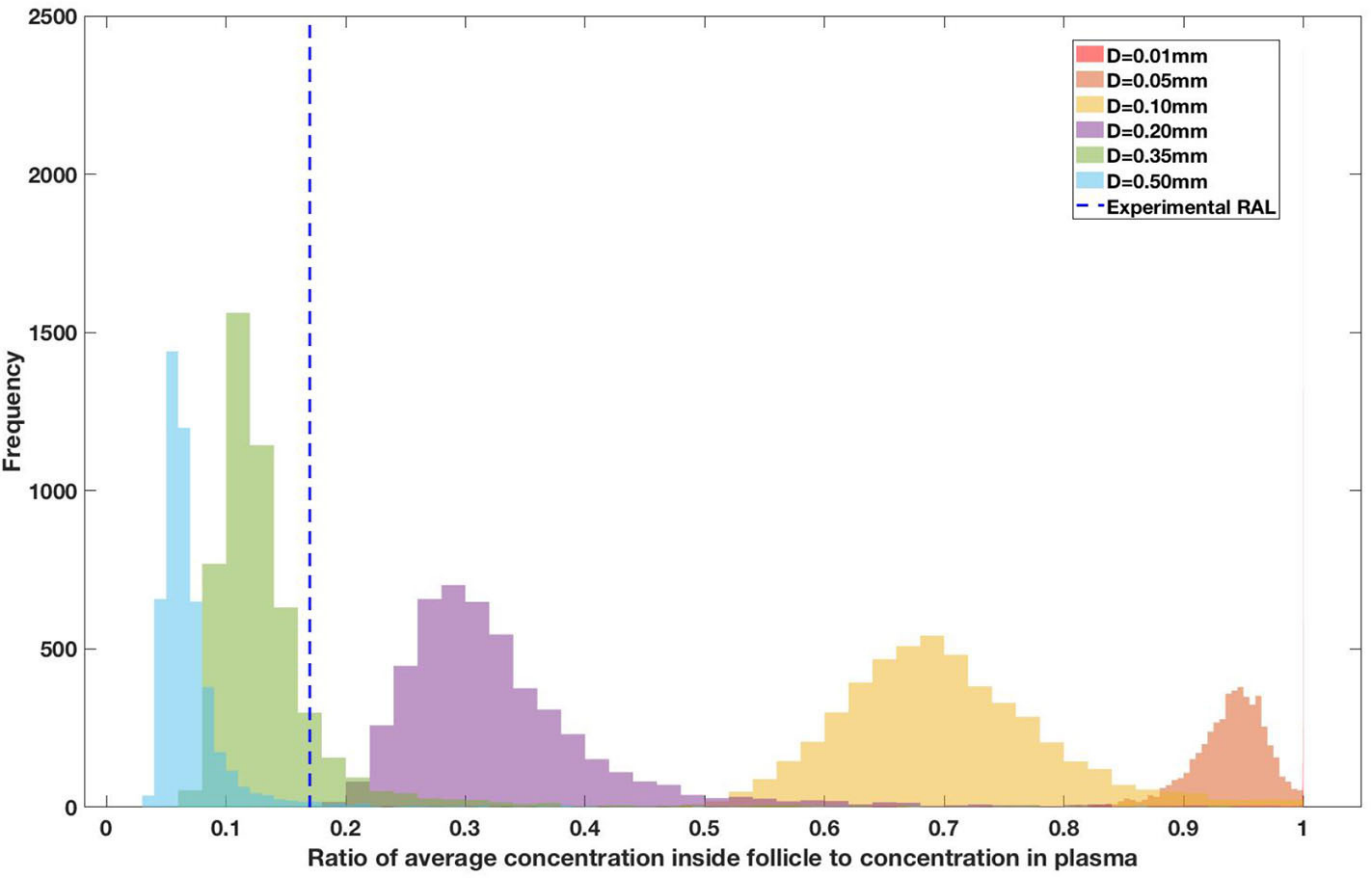
Concentration ratio (lobule/plasma) of Raltegravir, RAL (INSTI) with change in lobule diameter.

**Figure 11:**
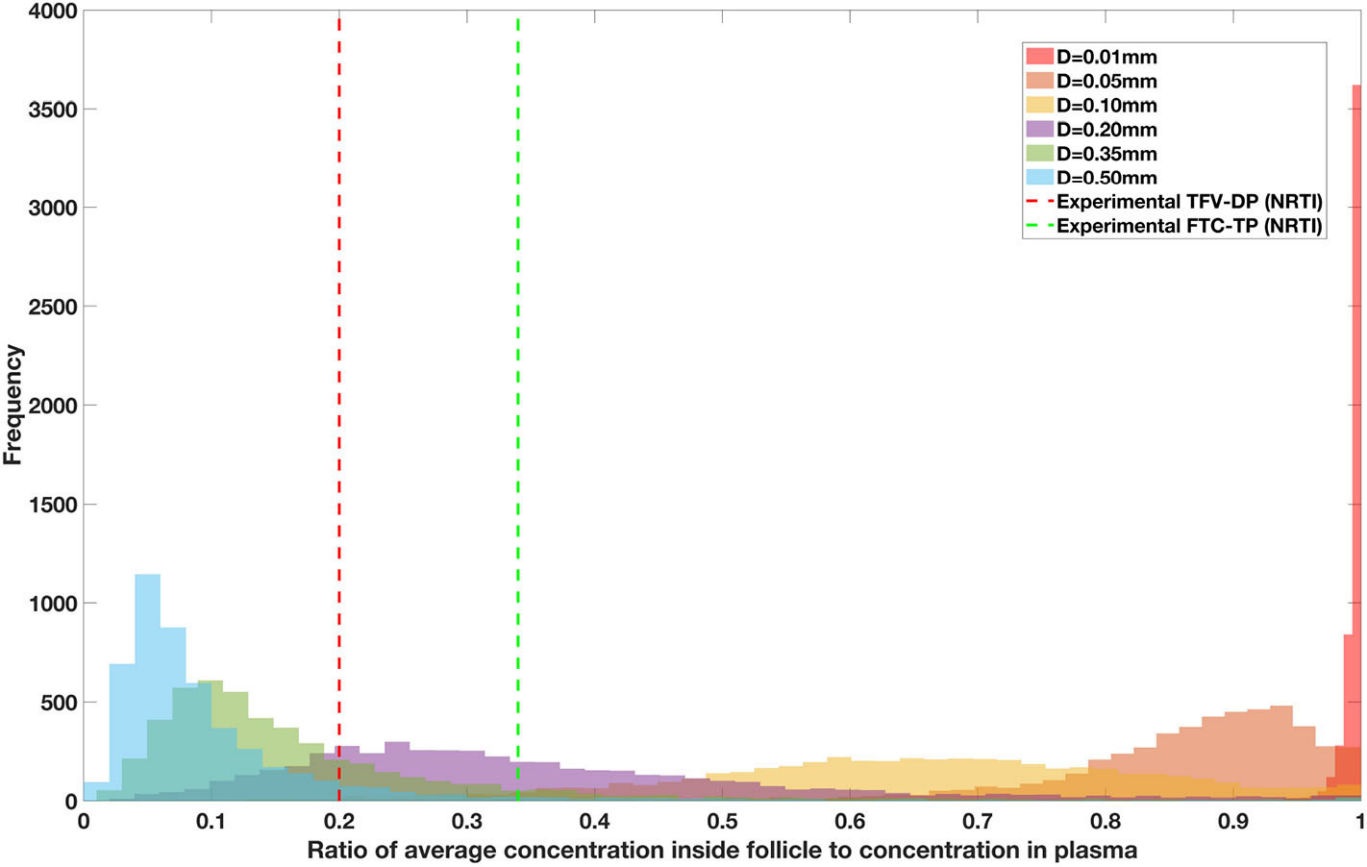
Concentration ratio (lobule/plasma) of Lamivudine, LMV (nRTI) with change in lobule diameter.

**Figure 12:**
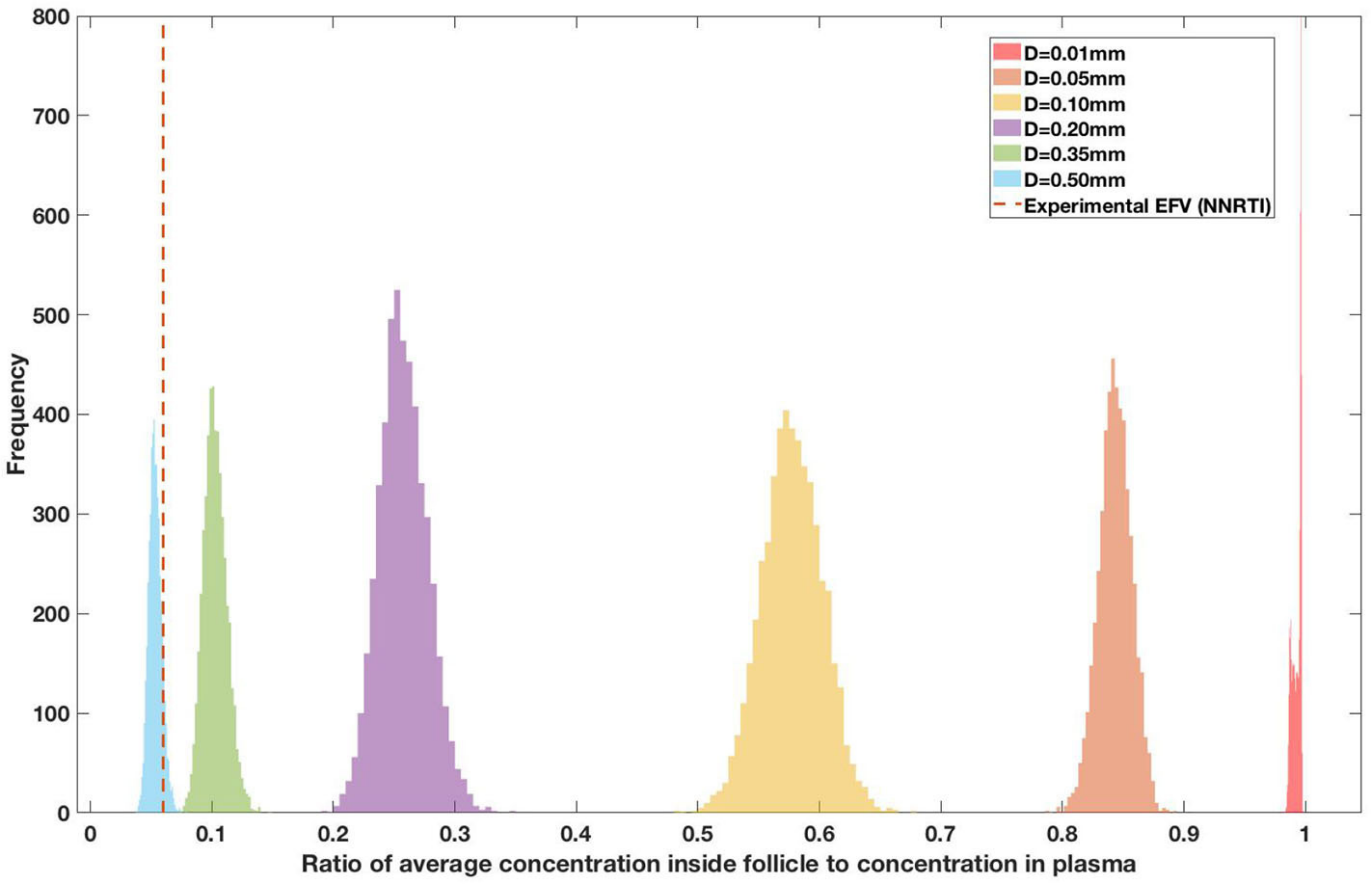
Concentration ratio (lobule/plasma) of Efarivenz, EFV (NNRTI) with change in lobule diameter.

Drug penetration for various anti-retroviral drugs in the lymphoid lobules has been evaluated using our integrated spatial dynamic pharmacokinetic model, following the Monte-Carlo methods described above. The three cART regimens simulated were NNN (tenofovir, lamivudine, efavirenz), NNP (tenofovir, efavirenz, ritonavir), and NNI (tenofovir, lamivudine, raltegravir). These three drug combinations were simulated on lymphoid lobules with diameters of 0.01, 0.05, 0.10, 0.20, 0.35, and 0.50 mm. For each combination of drug regimen and lobule size, 5,000 sets of parameters were randomly drawn from the parameter distributions described in Tables S1–S8. Intracellular drug penetration ratios (DPR) between the lymphoid lobule and the plasma were calculated for each drug. The intracellular drug concentration inside the lobule is evaluated by averaging the concentration over the entire volume of the lobule. Figures 8–12 show histograms of the predicted drug concentration ratio between lobule and plasma for the six different lobule sizes. Vertical dashed lines show experimentally measured ratios for drugs of the same class. The DPR consistently drops as the lobule size increases, though the strength of this effect varies from drug to drug. The posterior distributions depend on the uncertainty in the PK parameters for the individual drugs; lamivudine, in particular, has very broad posterior distributions due to a high published uncertainty in its PK parameters (Figure 11). The experimentally measured DPRs seem to correlate best with lobule sizes of approximately 0.2 mm in diameter, though this underestimates drug exclusion for efavirenz and ritonavir. A lobule diameter of 0.2 mm would correspond to a moderate state of inflammation consistent with treated HIV infection.

Assuming an average uninflamed lymphoid lobule to be of 0.2 mm in diameter, our model predicts median DPR between lobule and plasma of 10% for PI (Ritonavir, RTV), 25.40% for NNRTI (Efarivenz, EFV), 17.80% for nRTI (Tenofovir diphosphate, TFV-DP), 30.70% for nRTI (Lamivudine, LMV) and 27.67% for INSTI (Raltegravir, RAL). These results reproduce the experimentally reported median intracellular tissue (Lymph node) to plasma ratio values for nRTIs such as Tenofovir Diphosphate (TFV-DP) and Emtricitabine (FTC-TP) at 20 and 34%, respectively, INSTI such as Raltegravir (RAL) AT 17%, PI's such as Atazanavir (ATV) and Darunavir (DRV) at 0 and 1%, respectively and NNRTIs such as Efavirenz (EFV) at 6% (Fletcher et al., 2014). Lobules with diameters of 0.5 mm, which would correspond to extreme levels of inflammation, predicted median DPRs under 10% for all drugs. In the absence of inflammation (lobule diameter 0.1 mm or less), median predicted DPRs were over 50% for all drugs except ritonovir.

### 3.2. Sanctuary Site Formation vs. Treatment Combination

**Figure 13:**
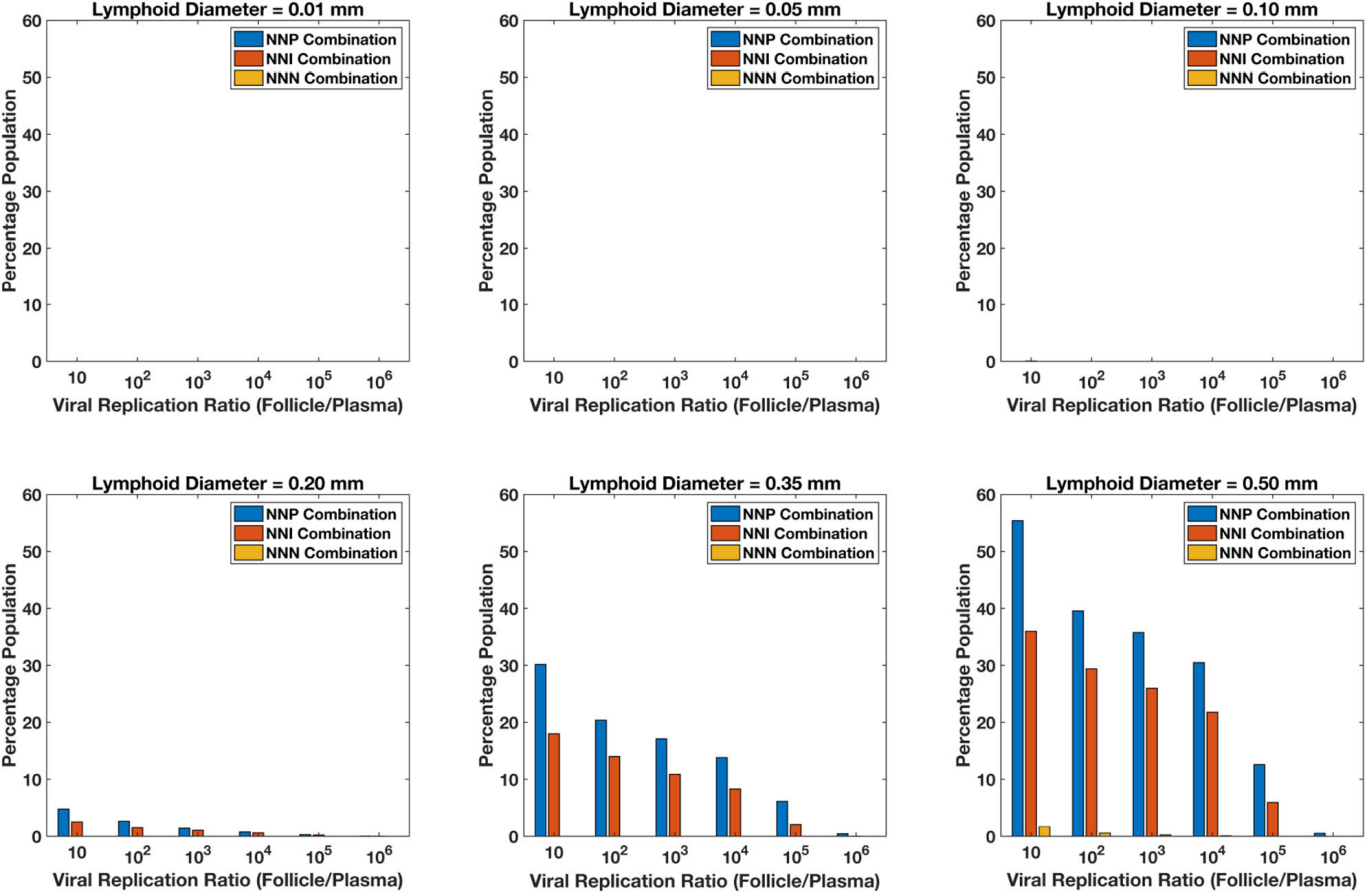
Percentage sanctuary site formation with varying treatment combinations and lymphoid lobule diameter.

For each of the 5,000 simulations for each drug regimen and lobule size combination described above, HIV dynamics were also simulated in the blood/lymph compartment as well as in the lobule. For each simulation, the fold increase in viral replication inside the lobule relative to the blood lymph compartment was measured once the dynamics reached steady-state. Figure 13 shows the percentage of the simulations with a fold increase of viral replication in the lobule at each order of magnitude relative to the blood, grouped by drug regimen. Our model predictions suggest no ongoing viral replication occurs in lobules with diameter 0.01, 0.05, and 0.10 mm during on treatment conditions across all treatment combinations. Less than 5% of the population showed a 10-fold increase in viral replication inside the sanctuary site compared to plasma levels for patients on treatment with NNP (Two nRTIs and a PI) and NNI (Two nRTIs and a INSTI) for lobule size of 0.2 mm in diameter. The proportion of population with ongoing viral replication inside the lobule increases with increase in diameter of the lobule for both NNP and NNI treatment conditions, with NNP always having a higher percentage of population with ongoing replication compared to NNI. Treatment combination with NNRTIs i.e., NNN (two nRTIs with a NNRTI) did not show any sanctuary site formation for lobule diameters less than 0.5 mm. However, the proportion of population with ongoing replication under NNN combination is still less than 2% for a lobule size as large as 0.5 mm.

Our simulations suggest that the chances of sanctuary site formation are higher under NNP treatment combination compared to the other combinations. This result is consistent with the predicted drug penetration results because PIs are the most excluded drugs compared to the other class of drugs. Even though INSTIs and NNRTIs have similar penetration levels inside the lobules, the proportion of population with sanctuary site formation is less in the case of NNRTI-containing regimens. This is likely due to the superior pharmacodynamic profile of NNRTIs, which have a low IC50 relative to their target dose and a higher Hill coefficient compared to INSTIs.

Our model predicts that sanctuary site formation while on cART is rare except under conditions where lymphoid lobules are very large. However, the proportion of population with sanctuary site formation increases with increase in lobule size, and NNP regimens are the most like to lead to sanctuary site formation.

## 4. Discussion

In our current study, we integrated our previously published HIV dynamics spatial compartmental model with a pharmacokinetic model to evaluate the drug penetration inside a lymphoid lobule. Our predictions on antiviral drug concentrations inside the lobule suggest that drug penetration decreases with increase in lymphoid lobule size, with less than 50% of the drug reaching the interior regions for lobule diameters greater than 0.10 mm. PIs are the least penetrative drugs compared to INSTIs and NNRTIs. Our model predictions on drug penetration were matched against previously published experimental observations for validation purposes (Fletcher et al., 2014). Drug penetration results for an average lymphoid lobule diameter of 0.2 mm match with the experimental drug penetration values. Our model does tend to underestimate the exclusion of PIs and NNRTIs compared to the experimental data. Our model incorporates only the most basic pharmacokinetic properties of the drugs in its transport model, and the two drugs in question have chemical properties that could significantly affect their transport rates across the lobule boundaries that are not captured in our model. Efavirenz is known to be unusually lipophilic, which will affect it transport rates across any plasma membrane boundary, and ritonavir is known to have a very high protein-bound fraction, which could significantly affect its transport rates across both the HEV and the lymphoid sinus boundaries. Future experiments could directly measure these transport rates. Furthermore, none of the experimental results contain information on the lobule size corresponding to their observed results. Since the drug penetration values are size-dependent as shown in our results, we suggest future experimental designs include determination of size and location for evaluating the drug penetration values experimentally.

The low drug penetration of PI results in a much higher proportion of virtual patients on NNP treatment regimens forming sanctuary sites compared to NNN and NNI combinations. Most patients, however, do not form sanctuary sites in our model. Formation of sanctuary sites (characterized by elevated viral replication in a treated patient) depends on the patient-specific drug transport dynamics, the PK/PD dynamics of the individual patient, the virus dynamics of the individual patient, and most strongly on the inflammation status of the lymphoid lobule in question. The limit of detection for increased viral activity in the lobule would probably be at least a 100-fold increase compared to the blood, and median parameter values never displayed this level of increase for any level of inflammation up to lobule diameters of 0.5 mm. Monte-Carlo studies exploring the range of parameter uncertainties revealed that sanctuary site behavior at the level of 100-fold increase does begin to emerge once lobules reach diameters of 0.2 mm, but only 3% of the population on NNP regimens and 2% of patients on NNI regimens would be expected to exhibit any sanctuary site activity at this level of inflammation. Increasing inflammation beyond this point does result in increased probability of sanctuary site formation, but lobules with diameters of 0.5 mm or larger likely represent pathological levels of lymphoid hyperplasia.

These predictions are broadly consistent with the clinical observations. The amount of viral replication in mono-nuclear cells inside lymph nodes is 10- to 100-fold greater, and the frequency of cells containing HIV DNA is 5- to 10-fold greater, than that in PBMC (Pantaleo et al., 1991, 1993). Furthermore, the absence of measurable viral load in the blood compartment does not rule out the possibility of ongoing low-level viral replication. It has been well-established that transport between the lymphoid sites and the blood is limited (Fletcher et al., 2014), which almost certainly limits the transport of infected cells and virus. Similarly, the lack of any observations on sequence evolution in HIV through experiments (Anderson et al., 2011; Evering et al., 2012) does not rule out the possibility of isolated ongoing replication; the small numbers of infected cells produced in the site, coupled with the limited transport between the site and the blood, mean that this would have to persist for a very long time to measurably influence the genetic distribution of the integrated HIV DNA in circulating cells. Furthermore, the low population incidence rates predicted by our model make it likely that this would be missed by all except the largest studies.

There is also some evidence of a positive feedback mechanism whereby viral activity in a lymphoid site causes physiological changes to the site that promote sanctuary site activity. Since the majority of the HIV infections are harbored in the paracortical site of the LN, an increase in traffic of CD4+ T cells to mount an immune response in the lymph node causes inflammation of the lymphoid lobule. As HIV infection progresses the histopathology of the LN changes toward hyperplasia in the beginning and eventually leading to follicular involution (Paiva et al., 1996; Cohen et al., 1997). As discussed above, increased lobule volume decreases anti-retroviral transport into the lobule, increases cell residence time, and enables localized viral replication. Marked collagen deposition in the paracortical T-cell zone of inguinal lymph nodes in HIV infected individuals has also been observed (Schacker et al., 2002). These observed changes in the architecture of the lymphatic tissue and the increase in size of the lobule due to immune activation might affect the penetration of anti-retroviral drugs during HIV infection. These effects are not captured in this model, but represent an avenue of future research.

## Data Availability Statement

The datasets generated for this study are available on request to the corresponding author.

## Author Contributions

AJ contributed to model development, code development, data generation and analysis, writing, and editing the manuscript. MP contributed to model development, data analysis, and editing the manuscript. RZ contributed to model development, data analysis, writing, and editing the manuscript.

## Conflict of Interest

RZ is named as an inventor on US Patent 9874563, Detecting and quantifying cryptic HIV replication. The remaining authors declare that the research was conducted in the absence of any commercial or financial relationships that could be construed as a potential conflict of interest.
