# Supplementary material for: An Integrated Spatial Dynamics—Pharmacokinetic Model Explaining Poor Penetration of Anti-retroviral Drugs in Lymph Nodes
Source: Front Bioeng Biotechnol. 2020 Jun 26;8:667. doi: 10.3389/fbioe.2020.00667 (PMC7333380; doi:10.3389/fbioe.2020.00667)
Supplement: Supplementary file 1 [file Data_Sheet_1.PDF]

Supplementary Material

1 SUPPLEMENTARY DATA

1.1 Virus dynamics (Model adapted from Cardozo et al., 2012)

Equations 1 to 3 describe HIV dynamics under various treatment combinations. Coefficients $\mathrm{u_{n1}}$, $\mathrm{u_{n2}}$, $\mathrm{u_i}$, $\mathrm{u_r}$ and $\mathrm{u_p}$ were used as switches to activate each individual drug NRTI1, NRTI2, INSTI, NNRTI and PI respectively. In order to activate NNP (two NRTIs and one PI) treatment combination, coefficients $\mathrm{u_{n1}}$, $\mathrm{u_{n2}}$ and $\mathrm{u_p}$ each are assigned a value of 1 while the rest of the coefficients are assigned 0. Similarly for NNI (two NRTIs and one INSTI), coefficients $\mathrm{u_{n1}}$, $\mathrm{u_{n2}}$ and $\mathrm{u_i}$ each are assigned a value of 1 while the rest of the coefficients are assigned 0. For NNN combination (two NRTIs and one NNRTI), coefficients $\mathrm{u_{n1}}$, $\mathrm{u_{n2}}$ and $\mathrm{u_r}$ each are assigned a value of 1 while the rest of the coefficients are assigned 0.

Uninfected/Target Cells (X)

$$\begin{aligned}\frac{\mathbf{dX_{(s)}}}{\mathbf{dt}} =& \lambda - d\mathbf{X}_{(s)} \\ & - \beta \mathbf{X}_{(s)} \mathbf{V}_{(s)} (1 - \epsilon_{NRTI1(s)} u_{n1})(1 - \epsilon_{NRTI2(s)} u_{n2})(1 - \epsilon_{INSTI(s)} u_i)(1 - \epsilon_{INSTI(s)} u_r) \\ & + \sum_{i \in \psi_s} \frac{D_{x\ i,s}}{l} \frac{A_{i,s}}{V_s} (\mathbf{X}_{(i)} - \mathbf{X}_{(s)})\end{aligned} \tag{S1}$$

Infected Cells (Y)

$$\begin{aligned}\frac{\mathbf{dY_{(s)}}}{\mathbf{dt}} =& \beta \mathbf{X}_{(s)} \mathbf{V}_{(s)} (1 - \epsilon_{NRTI1(s)} u_{n1})(1 - \epsilon_{NRTI2(s)} u_{n2})(1 - \epsilon_{INSTI(s)} u_i)(1 - \epsilon_{INSTI(s)} u_r) \\ & - a\mathbf{Y}_{(s)} + Y_e + \sum_{i \in \psi_s} \frac{D_{y\ i,s}}{l} \frac{A_{i,s}}{V_s} (\mathbf{Y}_{(i)} - \mathbf{Y}_{(s)})\end{aligned} \tag{S2}$$

Virus (V)

$$\frac{\mathbf{dV_{(s)}}}{\mathbf{dt}} = \gamma(1 - \epsilon_{PI(s)} u_p)\mathbf{Y}_{(s)} - \omega \mathbf{V}_{(s)} + \sum_{i \in \psi_s} \frac{D_{v\ i,s}}{l} \frac{A_{i,s}}{V_s} (\mathbf{V}_{(i)} - \mathbf{V}_{(s)}) \tag{S3}$$

1.2 Tenofovir Disoproxil Fumarate (TDF) - NRTI-1 (adapted from Dixit & Perelson., 2004)

Tenofovir Drug (T)

$$\frac{\mathbf{dT}}{\mathbf{dt}} = -k_{Ta} F_T \mathbf{T} \tag{S4}$$

Tenofovir Plasma (T_p)

$$\frac{\mathbf{dT_{P(s)}}}{\mathbf{dt}} = \left(\frac{k_{Ta} F_T \mathbf{T}}{V_{T_P}}\right)^* - k_{Te}\mathbf{T_{P(s)}} + \sum_{i\in\psi_s} \frac{D_{Tp\ i,s}}{l}\frac{A_{i,s}}{V_s}(\mathbf{T_{P(i)}} - \mathbf{T_{P(s)}}) \tag{S5}$$

* term only exists for s=1 compartment

Tenofovir Intracellular (T_c)

$$\begin{aligned}\frac{\mathbf{dT_{C(s)}}}{\mathbf{dt}} =& k_{Tacell}((1-f_{BT})H_T\mathbf{T_{P(s)}} - \mathbf{T_{C(s)}}) - k_{Tecell}\mathbf{T_{C(s)}} - k_{1f}\mathbf{T_{C(s)}} \\ &+ k_{1b}\mathbf{T_{CMP(s)}} + \sum_{i\in\psi_s} \frac{D_{x\ i,s}}{l}\frac{A_{i,s}}{V_s}(\mathbf{T_{C(i)}} - \mathbf{T_{C(s)}})\end{aligned} \tag{S6}$$

Tenofovir (monophosphate) Intracellular (T_{cmp})

$$\begin{aligned}\frac{\mathbf{dT_{CMP(s)}}}{\mathbf{dt}} =& k_{1f}\mathbf{T_{C(s)}} - k_{1b}\mathbf{T_{CMP(s)}} + k_{2b}\mathbf{T_{CDP(s)}} - k_{2f}\mathbf{T_{CMP(s)}} \\ &- k_{Tecell}\mathbf{T_{CMP(s)}} + \sum_{i\in\psi_s} \frac{D_{x\ i,s}}{l}\frac{A_{i,s}}{V_s}(\mathbf{T_{CMP(i)}} - \mathbf{T_{CMP(s)}})\end{aligned} \tag{S7}$$

Tenofovir (diphosphate) Intracellular (T_{cdp})

$$\begin{aligned}\frac{\mathbf{dT_{CDP(s)}}}{\mathbf{dt}} =& k_{2f}\mathbf{T_{CMP(s)}} - k_{2b}\mathbf{T_{CDP(s)}} - k_{Tecell}\mathbf{T_{CDP(s)}} \\ &+ \sum_{i\in\psi_s} \frac{D_{x\ i,s}}{l}\frac{A_{i,s}}{V_s}(\mathbf{T_{CDP(i)}} - \mathbf{T_{CDP(s)}})\end{aligned} \tag{S8}$$

Tenofovir Efficacy ($\epsilon_{\mathrm{NRTI1}}$)

$$\epsilon_{NRTI1(S)} = \left(\frac{\mathbf{T_{CDP(s)}}^{n_T}}{IC50_T{}^{n_T} + \mathbf{T_{CDP(s)}}^{n_T}}\right) \tag{S9}$$

1.3 Lamivudine (3TC) -NRTI2 (adapted from Hurwitz et al., 2007)

Lamivudine Plasma (L_P)

$$\begin{aligned}\frac{\mathbf{dL_{P(s)}}}{\mathbf{dt}} =& Inp^* - k_{EP}\mathbf{L_{P(s)}} - (k_{PT}\mathbf{L_{P(1)}})^{\#} + (k_{TP}\mathbf{L_{DT(1)}})^{\#} \\ &+ \sum_{i\in\psi_s} \frac{D_{Lp\ i,s}}{l}\frac{A_{i,s}}{V_s}(\mathbf{L_{P(i)}} - \mathbf{L_{P(s)}})\end{aligned} \tag{S10}$$

* Zero order drug input is only to the first compartment (s=1) of the Lamivudine Plasma i.e. $L_{P(1)}$. Rest of the compartments pertaining to Lamivudine Plasma $L_{P(S)}$ have no access to the input.
# These terms only exist for first compartment of Lamivudine Plasma connecting to the dead tissue. These terms do not exist for other compartments (s>1) corresponding to Lamivudine Plasma.

Lamivudine Deep Tissue (L_{DT})

$$\frac{\mathbf{dL_{DT(1)}}}{\mathbf{dt}} = k_{PT}\mathbf{L_{p(1)}} + k_{TP}\mathbf{L_{DT(1)}} \tag{S11}$$

Lamivudine Intracellular Concentration (L_C)

$$\frac{\mathbf{dL_P}}{\mathbf{dt}} = \frac{\mathbf{dL_C}}{\mathbf{dt}} \tag{S12}$$

Lamivudine Total Nucleotides Intracellular (L_{TotN})

$$\begin{aligned}\frac{\mathbf{dL_{TotN(s)}}}{\mathbf{dt}} =& \left(\frac{V_{max}}{\mathbf{L_{C(s)}} + K_m}\right)\mathbf{L_{C(s)}} + k_{M-CMP}\mathbf{M_{(s)}} \\ & - R_{\frac{CMP}{TotN}} k_{CMP-C}\mathbf{L_{TotN(s)}} \\ & - R_{\frac{CMP}{TotN}} R_{\frac{CTP}{CDP}} R_{\frac{CDP}{CMP}} K_{TCP-M}\mathbf{L_{TotN(s)}} \\ & + \sum_{i \in \psi_s} \frac{D_{x\ i,s}}{l}\frac{A_{i,s}}{V_s}(\mathbf{L_{TotN(i)}} - \mathbf{L_{TotN(s)}})\end{aligned} \tag{S13}$$

Intracellular Metabolite (M)

$$\frac{\mathbf{dM_{(s)}}}{\mathbf{dt}} = R_{\frac{CMP}{TotN}} R_{\frac{CTP}{CDP}} R_{\frac{CDP}{CMP}} K_{TCP-M}\mathbf{L_{TotN(s)}} - k_{M-CMP}\mathbf{M_{(s)}} \tag{S14}$$

Lamivudine Mono-phosphate (L_{CMP})

$$\mathbf{L_{CMP(s)}} = R_{\frac{CMP}{TotN}}\mathbf{L_{TotN(s)}} \tag{S15}$$

Lamivudine Di-phosphate (L_{CDP})

$$\mathbf{L_{CDP(s)}} = R_{\frac{CDP}{CMP}}\mathbf{L_{CMP(s)}} \tag{S16}$$

Lamivudine Tri-phosphate (L_{CTP})

$$\mathbf{L_{CTP(s)}} = R_{\frac{CTP}{CDP}}\mathbf{L_{CDP(s)}} \tag{S17}$$

1.4 Ritonavir (R) - PI (adapted from Dixit & Perelson., 2004)

Ritonavir Drug (R)

$$\frac{\mathbf{dR}}{\mathbf{dt}} = -k_{pa}F_p\mathbf{R} \tag{S18}$$

Ritonavir Plasma (R_P)

$$\begin{aligned}\frac{\mathbf{dR_{P(s)}}}{\mathbf{dt}} =& -\left(\frac{k_{pa}F_p\mathbf{R}}{V_{PP}}\right)^* - k_{pe}\mathbf{R_{P(s)}} - k_{pacell}\mathbf{R_{P(S)}} + k_{pecell}\mathbf{R_C}(\mathbf{s}) \\ &+ \sum_{i\in\psi_s}\frac{D_{PI\ i,s}}{l}\frac{A_{i,s}}{V_s}(\mathbf{R_{P(i)}} - \mathbf{R_{P(s)}})\end{aligned} \tag{S19}$$

* term exists only for first compartment corresponding to Ritonavir Plasma ($R_{P(1)}$)

Ritonavir Intracellular (R_C)

$$\frac{\mathbf{dR_{C(s)}}}{\mathbf{dt}} = k_{pacell}\mathbf{R_{P(S)}} - k_{pecell}\mathbf{R_C}(\mathbf{s}) + \sum_{i\in\psi_s}\frac{D_{x\ i,s}}{l}\frac{A_{i,s}}{V_s}(\mathbf{R_{C(i)}} - \mathbf{R_{C(s)}}) \tag{S20}$$

1.5 Raltegravir (RL) - INSTI (adapted from Arab-Alameddine et al., 2011)

Raltegravir Drug (RL)

$$\frac{\mathbf{dRL}}{\mathbf{dt}} = -k_{RLa}\mathbf{RL} \tag{S21}$$

Raltegravir Plasma (RL_P)

$$\begin{aligned}\frac{\mathbf{dRL_{P(s)}}}{\mathbf{dt}} =& \left[k_{RLa}\mathbf{RL} - \left(\frac{Q/F}{V_P/F}\right)\mathbf{RL_{P(1)}} + \left(\frac{Q/F}{V_P/F}\right)\mathbf{RL_{PH}}\right]^* - \left(\frac{Cl/F}{V_P/F}\right)\mathbf{RL_{P(s)}} \\ &+ \sum_{i\in\psi_s}\frac{D_{RL\ i,s}}{l}\frac{A_{i,s}}{V_s}(\mathbf{RL_{P(i)}} - \mathbf{RL_{P(s)}})\end{aligned} \tag{S22}$$

* terms exist only for first compartment i.e. (s=1) since they interact only with the outermost spherical compartment of Raltegravir Plasma concentrations.

Raltegravir Pheripheral (RL_{PH})

$$\frac{\mathbf{dRL_{PH}}}{\mathbf{dt}} = \left(\frac{Q/F}{V_{PH}/F}\right)\mathbf{RL_{P(1)}} - \left(\frac{Q/F}{V_{PH}/F}\right)\mathbf{RL_{PH}} \tag{S23}$$

Raltegravir Intracellular ($\mathrm{RL_C}$)

$$\begin{aligned}\frac{\mathbf{dRL_{C(s)}}}{\mathbf{dt}} =& k_{RLin}\mathbf{RL_{P(s)}} - k_{RLout}\mathbf{RL_{C(s)}} \\ &+ \sum_{i\in\psi_s} \frac{D_{x\ i,s}}{l}\frac{A_{i,s}}{V_s}(\mathbf{RL_{C(i)}} - \mathbf{RL_{C(s)}})\end{aligned} \tag{S24}$$

1.6 Efavirenz (EFV) - NNRTI (adapted from Habtewold et al., 2017)

Efavirenz Drug (E)

$$\frac{\mathbf{dE}}{\mathbf{dt}} = -k_{Ea}\mathbf{E} \tag{S25}$$

Efavirenz Plasma ($\mathrm{E_P}$)

$$\begin{aligned}\frac{\mathbf{dE_{P(s)}}}{\mathbf{dt}} =& \left(\frac{k_{Ea}\mathbf{E}}{V_E}\right)^{*} - k_{PH}\mathbf{E_{P(s)}} - k_{Ein}\mathbf{E_{P(s)}} + \left(\frac{V_{Emax}\mathbf{E_{C(s)}}}{K_{Em} + \mathbf{E_{C(s)}}}\right) \\ &+ \sum_{i\in\psi_s} \frac{D_{E\ i,s}}{l}\frac{A_{i,s}}{V_s}(\mathbf{E_{P(i)}} - \mathbf{E_{P(s)}})\end{aligned} \tag{S26}$$

* term exists only for the first compartment i.e. (s=1) since they interact only with the outermost spherical compartment of Efavirenz Plasma concentrations.

Efavirenz Intracellular ($\mathrm{E_C}$)

$$\begin{aligned}\frac{\mathbf{dE_{C(s)}}}{\mathbf{dt}} =& k_{Ein}\mathbf{E_{P(s)}} - \left(\frac{V_{Emax}\mathbf{E_{C(s)}}}{K_{Em} + \mathbf{E_{C(s)}}}\right) \\ &+ \sum_{i\in\psi_s} \frac{D_{x\ i,s}}{l}\frac{A_{i,s}}{V_s}(\mathbf{E_{C(i)}} - \mathbf{E_{C(s)}})\end{aligned} \tag{S27}$$

2 SUPPLEMENTARY TABLES

2.1 Table S1: Parameter values for HIV dynamics from Luo et al. (2012)

Parameter	Units	Value
Target cell production rate (λ)	$\log_{10}$ ($cells$/μLxday)	(1.54,2.88)
Target cell death rate (d)	$\log_{10}(1/day)$	(-1.35,-0.34)
Density dependent infection rate (β)	$\log_{10}(mL/copiesxday)$	(-5.78,-5.23)
Infected cell death rate (δ)	$\log_{10}(1/day)$	(-0.76,0.42)
Virus production rate (p)	$\log_{10}$($copies.\mu$L/$cells$ x mL x day)	(3.39,4.00)
Virus clearance rate (c)	$\log_{10}(1/day)$	1.27

Above parameters were drawn from multivariate posterior distributions published in Luo et al., 2012

2.2 Table S2: Adjusted Drug Diffusion Coefficient Values estimated using Einstein Stokes Equation

**Drug**	**MW** (g/mol)	**Drug Density** (g/m^3)	**Stokes Radius** (m)	**Diffusive Coefficient** (mm^2/sec)	**Adjusted Value** (mm^2/sec)
Tenofovir (NRTI1)	287.216	1800000	3.9837E-10	27.1840042	1.197
Lamivudine (NRTI2)	229.26	1700000	3.76647E-10	28.75184254	1.266
Ritonavir (PI)	720.948	1200000	6.19749E-10	17.47368779	0.769
Efarivenz (NNRTI)	315.676	1500000	4.36876E-10	24.78803124	1.091
Raltegravir (INSTI)	444.423	1500000	4.89639E-10	22.1168725	0.974

2.3 Table S3: Estimated diffusion coefficient between blood/lymph and follicle

Parameter	Definition	Units	Value
$D_{x1,2}$/l	Estimated T-cells permeability between the blood and follicle	mm/day	1/300
$D_{v1,2}$/l	Estimated HIV permeability between the blood and follicle	mm/day	10^{-8}
$D_{T1,2}$	Estimated Tenofovir (TFV) diffusion coefficient between the blood and follicle	mm^2/day	4.41E-04
$D_{L1,2}$	Estimated Lamivudine (3TC) diffusion coefficient between the blood and follicle	mm^2/day	4.67E-04
$D_{R1,2}$	Estimated Raltegravir (RAL) diffusion coefficient between the blood and follicle	mm^2/day	3.59E-04
$D_{E1,2}$	Estimated Efavirenz (EFV) diffusion coefficient between the blood and follicle	mm^2/day	4.04E-04
$D_{RT1,2}$	Estimated RTV permeability between the blood and follicle	mm^2/day	2.81E-04

2.4 Table S4: Tenofovir Diphosphate (TFV-DP) Pharmacokinetic Parameters

Parameter	Definition	Units	Nominal Value	95% CI	Assumed Distribution
k_{Ta}	Drug adsorption rate constant from gut to plasma (Baheti et al., 2011)	$1/day$	19.72	[12.00 , 27.44]	Normal
F_r	Bioavailability of TFV (Dixit & Perelson, 2004)	—-	0.39	[0.33 , 0.44]	Normal
V_{Tp}	Volume of Distribution in blood for TFV (Baheti et al., 2011)	ml	87360	[51845 , 122727]	Normal
k_{Te}	Elimination rate constant from plasma (Baheti et al., 2011)	$1/day$	4.74	[3.14 , 7.64]	Compound
k_{Tacell}	Rate constant for transfer of TFV from extracellular to intracellular space (Dixit & Perelson, 2004)	$1/day$	24000	[19353 , 28718]*	Normal
k_{Tecell}	Rate constant for transfer of TFV from intracellular to extracellular space (Dixit & Perelson, 2004)	$1/day$	1.1	[0.90 , 1.29]*	Normal
k_{1f}	Rate constant for the forward reaction (TFV to TFV-MP) (Dixit & Perelson, 2004)	$1/day$	9.6	[7.83 , 11.36]*	Normal
k_{1b}	Rate constant for the backward reaction (TFV-MP to TFV) (Dixit & Perelson, 2004)	$1/day$	30.3	[24.38 , 36.16]*	Normal
k_{2f}	Rate constant for the forward reaction (TFV-MP to TFV-DP) (Dixit & Perelson, 2004)	$1/day$	270.7	[217.48 , 323.37]*	Normal
k_{2b}	Rate constant for the backward reaction (TFV-DP to TFV-MP) (Dixit & Perelson, 2004)	$1/day$	95.5	[75.94 , 115.11]*	Normal
IC_{50T}	Inhibition Coefficient (TFV) (Dixit & Perelson, 2004)	mg/ml	0.54	[0.012 , 3.141]	Lognormal
n_T	Hill coefficient for Tenofovir (Shen et al., 2008)	—-	0.97	—-	Constant

* assumed standard deviations (10% of the nominal value) when no information is available.

2.5 Table S5:Lamivudine Triphosphate (3TC-TP) Pharmacokinetic Parameters

Parameter	Definition	Units	Nominal Value	95 % CI	Assumed Distribution
CL/F	NRTI (Lamivudine) drug adsorption rate constant from gut to plasma (Hurwitz et al.,2007)	L/h	47.8	[9.89 , 89.97]	Normal
V_1/F	Effective volume of distribution for 3TC in plasma compartment (Hurwitz et al.,2007)	L	197	[30.50 , 400.21]	Normal
k_{pt}	Rate constant for transfer of NRTI (3TC) from Plasma to Dead Tissue (Hurwitz et al.,2007)	1/h	0.061	[0.0057 , 0.1553]	Normal
k_{tp}	Rate constant for transfer of NRTI (3TC) from Dead Tissue to Plasma (Hurwitz et al.,2007)	1/h	0.06	[0.0062 , 0.1394]	Normal
$V_{m,L}$	Maximum rate of conversion of cellular 3TC to 3TC-MP (Hurwitz et al.,2007)	pmol/10^6 cells/h	1.95	[0.91 , 10.62]	Normal
$K_{m,L}$	Average Michaelis Menten constant for Phosphorylation of 3TC to 3TC-MP (Hurwitz et al.,2007)	pmol/10^6 cells	0.58	[0.078 , 1.209]	Normal
$k_{Lcmp-Lc}$	Michaelis Menten constant for 3TC (Hurwitz et al.,2007)	1/h	2.02	[1.62 , 2.41]*	Normal
k_{tp-m}	Rate constant for transfer of 3TCMP to 3TC (Hurwitz et al.,2007)	1/h	0.16	[0.14 , 0.17]*	Normal
k_{m-mp}	Rate constant for conversion of Metabolite (M) to 3TCMP (Hurwitz et al.,2007)	1/h	0.46	[0.38 , 0.53]*	Normal
$R_{DP/MP}$	Ratio of 3TCDP/3TCMP (Hurwitz et al.,2007)	—-	6.65	[5.48 , 7.82]*	Normal
$R_{TP/DP}$	Ratio of 3TCTP/3TCDP (Hurwitz et al.,2007)	—-	1.02	[0.82 , 1.21]*	Normal
IC_{50L}	Inhibitory Coefficient (3TC) (Hurwitz et al.,2007)	μM	5.42	[3.83 , 7.65]	Lognormal
n_L	Hill Coefficient (3TC) (Shen et al., 2008)	—-	1.18	—-	Constant

K_{EP} (Rate of 3TC elimination from Plasma) = (CL/F)/(V_1/F)

$R_{TP/MP}$ = $R_{TP/DP}$*$R_{DP/MP}$

R$_{CMP/Total3TC}$ $= (1/1+$R$_{DP/MP}$+ $R_{TP/MP})$

* assumed standard deviations (10% of the nominal value) when no information is available.

2.6 Table S6: Raltegravir (RAL) Pharmacokinetic Parameters

Parameter	Definition	Units	Nominal Value	95 % CI	Assumed Distribution
k_{Ra}	drug adsorption rate constant from gut to plasma (Arab-Alameddine et al.,2012)	1/h	0.21	0.14-0.3	Normal
V_{Rp}	Volume of distribution for central compartment i.e. plasma (Arab-Alameddine et al.,2012)	L	223	65.6-481	Normal
cl_i	Clearance from central compartment (Arab-Alameddine et al.,2012)	L/h	60.2	27.6-148.9	Normal
q_i	Intercompartmental clearance (Arab-Alameddine et al.,2012)	L/h	8.5	2.6-20	Normal
V_{Rph}	Volume of distribution for peripheral compartment (Arab-Alameddine et al.,2012)	L	113	33.1-313	Normal
K_{Rin}	Rate constant for transfer of RAL from extracellular to intracellular space	1/h	0.1956$^{\#}$	[0.17 , 0.21]*	Normal
K_{Rout}	Rate constant for transfer of RAL from intracellular space to extracellular space	1/h	3.69$^{\#}$	[3.10 , 4.27]*	Normal
IC_{50R}	Inhibitory Coefficient (RAL)(Shen et al., 2008)	nm	14.9	[11.33 , 18.42]	Normal
n_R	Hill Coefficient (RAL) (Shen et al., 2008)	—-	1.1	—-	Constant

K_{Rin} and K_{Rout} have been estimated using the C_{cell} (Intracellular concentration) and C_{tot} (total plasma concentration) ratio of 0.053. (Fayet Mello et al., 2011)

* assumed standard deviations (10% of the nominal value) when no information is available.

2.7 Table S7: Ritonavir (RTV) Pharmacokinetic Parameters

**Parameter**	**Definition**	**Units**	**Nominal Value**	**95 % CI**	**Assumed Distribution**
k_{pa}	RTV drug adsorption rate constant from gut to plasma	1/day	19.71	[11.94 , 27.37]	Normal
V_{RTp}	Effective volume of distribution in blood for RTV (Dixit & Perelson, 2004)	ml	28000	[20153 , 35821]	Normal
k_{pe}	RTV elimination rate constant from plasma	1/day	9.92	[4.28 , 20.06]	Normal
k_{Pacell}	Rate constant for transfer of RTV from extracellular to intracellular space (Dixit & Perelson, 2004)	1/day	5.2	[4.21 , 6.19]*	Normal
k_{Pecell}	Rate constant for transfer of RTV from intracellular space to extracellular space (Dixit & Perelson, 2004)	1/day	10000	[9803 , 10195]*	Normal
F_p	Bioavailability of RTV (Dixit & Perelson, 2004)	—-	1	—-	Constant
f_{bp}	Protein binding of RTV (Dixit & Perelson, 2004)	—-	0.99	—-	Constant
H_p	Partition coefficient of RTV (Dixit & Perelson, 2004)	—-	0.052	—-	Constant
IC_{50RT}	Inhibitory Coefficient (RTV) (Dixit & Perelson, 2004)	mg/ml	$9 * 10^{-7}$	[2.12e-07,2.51e-06]	Lognormal
n_{RT}	Hill Coefficient (RTV)	—-	1	—-	Constant

* assumed standard deviations (10% of the nominal value) when no information is available.

2.8 Table S8: Efarivenz (EFV) Pharmacokinetic Parameters

Parameter	Definition	Units	Nominal Value	95 % CI	Assumed Distribution
k_{Ea}	EFV drug adsorption rate constant from gut to plasma (Habtewold et al., 2017)	1/h	0.2	[0.16 , 0.23]*	Normal
V_{Ep}	Effective volume of distribution in plasma for EFV (Habtewold et al., 2017)	L	100	[89.55 , 110.58]	Normal
V_{Epbmc}	Effective volume of distribution in PBMC for EFV (Habtewold et al., 2017)	L	210	[155.93 , 263.49]	Normal
k_{Ein}	Rate constant for transfer of EFV from Plasma to PBMC (Habtewold et al., 2017)	1/h	0.32	[0.26 , 0.38]	Compound
k_{Ee}	Elimination rate constant for Efarivenz from plasma (Habtewold et al., 2017)	1/h	0.10	[0.08 , 0.13]	Compound
V_{me}	Maximum rate for transfer of Efarivenz from PBMC to Plasma (Habtewold et al., 2017)	mg/ml/h	4400e-06	[4355e-06 , 4444e-06]	Normal
K_{me}	Average Michaelis Menten constant for transfer of Efarivenz from PBMC to Plasma (Habtewold et al., 2017)	mg/ml	710e-06	[664e-06 , 756e-06]	Normal
IC_{50E}	Inhibitory Coefficient (EFV) (Shen et al., 2008)	nm	5.4	[3.63 , 7.16]	Normal
n_E	Hill Coefficient (EFV) (Shen et al., 2008)	—-	1.69	—-	Constant

* assumed standard deviations (10% of the nominal value) when no information is available.
